# Supplementary material for: Dispersible hydrogel force sensors reveal patterns of solid mechanical stress in multicellular spheroid cultures
Source: Nat Commun. 2019 Jan 11;10:144. doi: 10.1038/s41467-018-07967-4 (PMC6329783; doi:10.1038/s41467-018-07967-4)
Supplement: Supplementary file 1 — Supplementary Information [file 41467_2018_7967_MOESM1_ESM.docx]

**Supplemental Figures.**


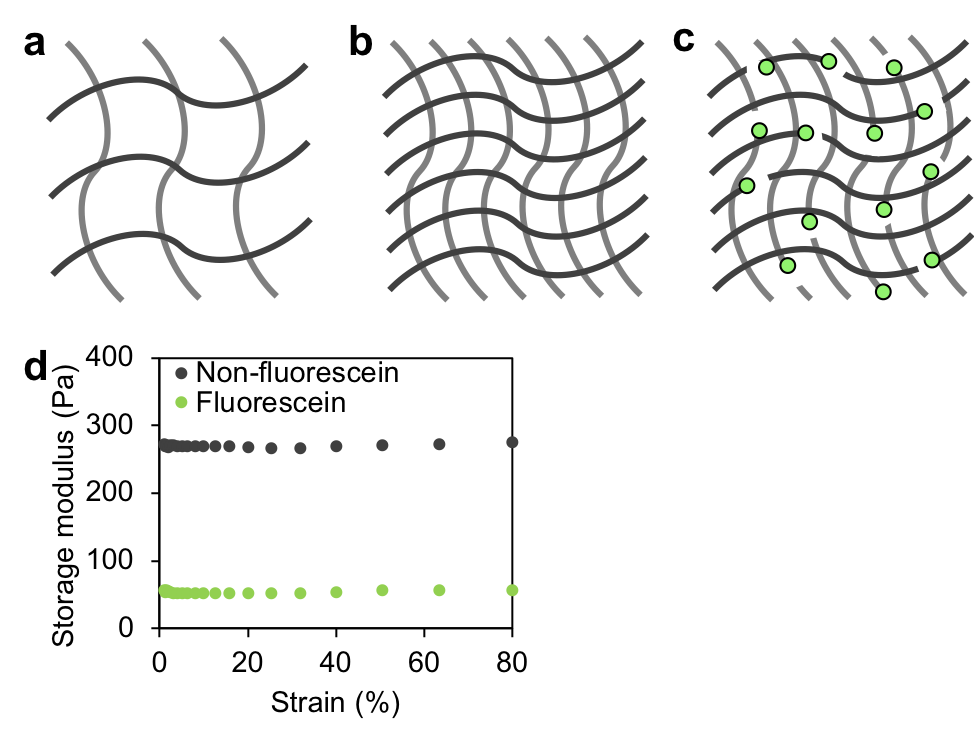


**Supplementary Figure 1.** Polyacrylamide matrices that are (**a**) low polymer content and soft; (**b**) high polymer content and stiff; and (**c**) high polymer content, but reduced stiffness due to incorporation of fluorescein o-methacrylate, a chain-terminating monomer. (**d**) Bulk shear rheology on polyacrylamide hydrogels polymerized with fluorescein o-methacrylate monomers demonstrate that for 100 μg/mL of fluorescein methacrylate, the stiffness of polyacrylamide matrices are reduced by five-fold compared to non-fluorescent hydrogels. The modified hydrogels retain their mechanical modulus properties over a large deformation range.


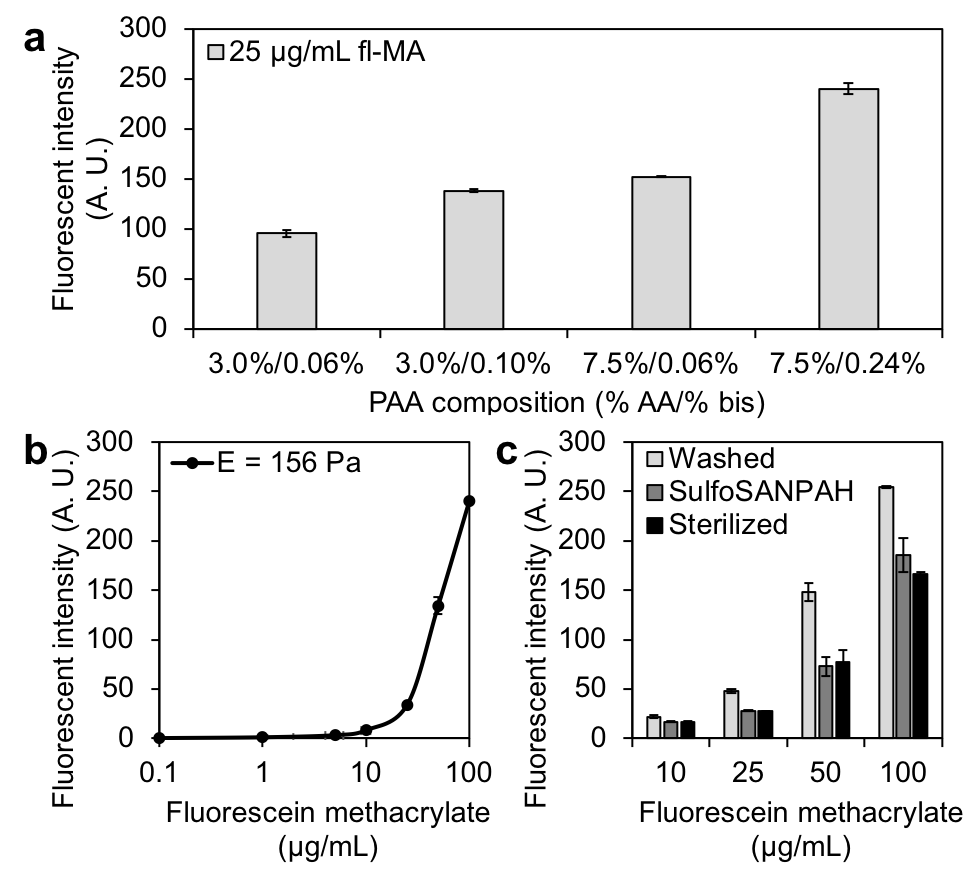


**Supplementary Figure 2.** Characterization of MSG fluorescence through co-polymerization with fluorescein methacrylate (fMA) monomers. Hydrogel fluorescent intensity depends on (**a**) MSG modulus, and (**b**) fMA concentration, as more polymerized fluorescent monomer results in higher fluorescent intensity. (**c**) Surface functionalization and sterilization steps further reduce the fluorescent intensity after the hydrogel is washed thoroughly. Data reported as mean ± standard deviation. These results indicate that fluorescein methacrylate is incorporated stably into the polymer backbone, allowing persistent labelling during cell culture experiments.


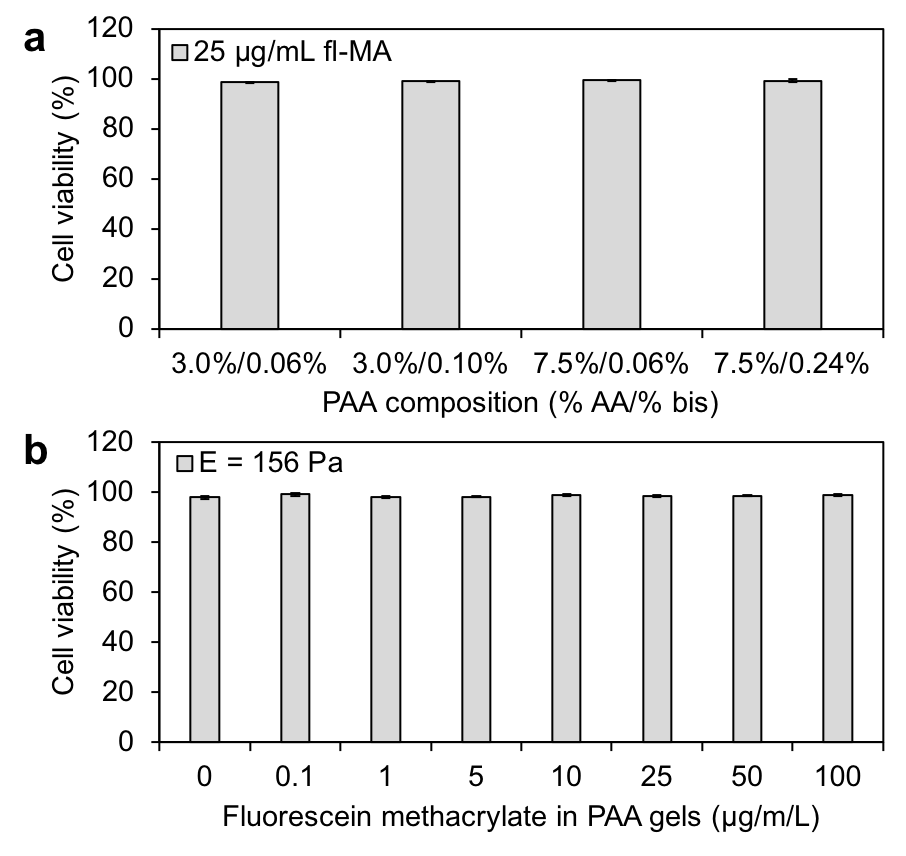


**Supplementary Figure 3.** Confirmation of cell viability in polyacrylamide hydrogels modified with fluorescein methacrylate monomers. HS-5 human bone marrow fibroblast cells cultured on polyacrylamide hydrogel formulations remained viable after 24 hours independent of (**a**) hydrogel modulus, and (**b**) fluorescent methacrylate concentration within the hydrogel. Data reported as mean ± standard deviation.

**
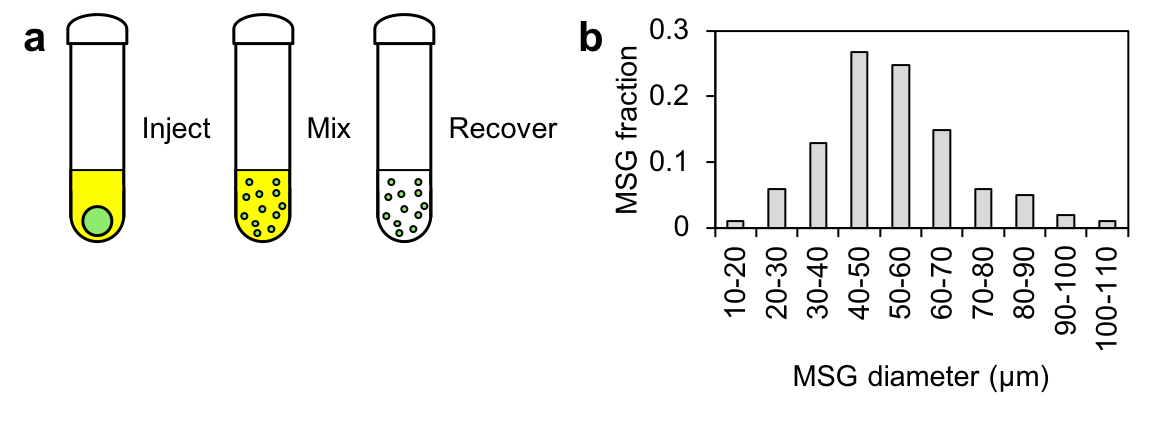
**

**Supplementary Figure 4.** Production and characterization of polydisperse hydrogel MSGs. (**a**) A two-phase oil/water immiscible system was used for the facile fabrication of hydrogel microspheres. Polyacrylamide pre-polymer with fluorescein methacrylate monomers were mechanically dispersed in an immiscible kerosene phase and allowed to polymerize under gentle stirring. (**b**) Size distribution of recovered microspheres indicate normal distribution of sizes with a mean diameter of ~50 μm.


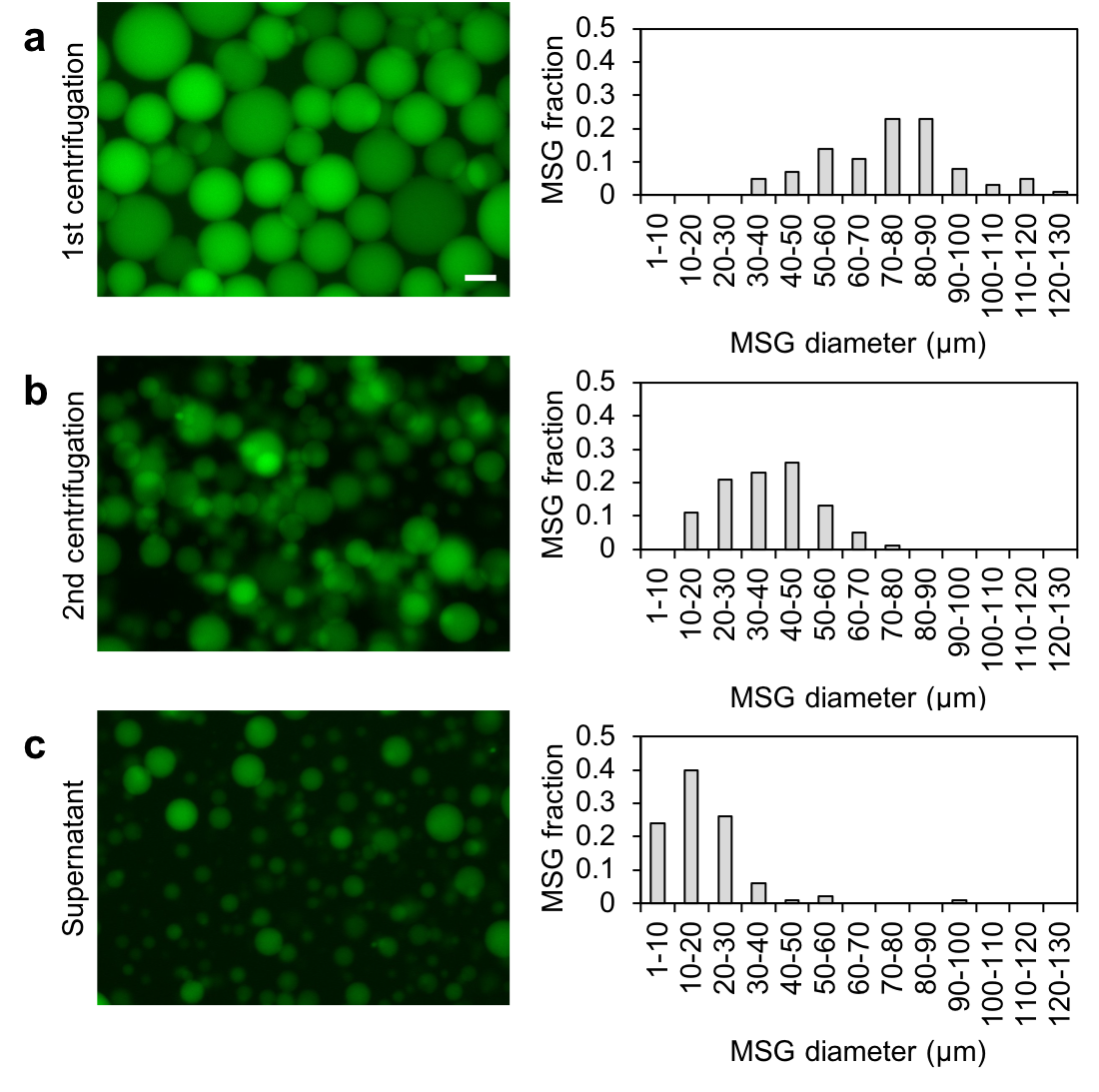


**Supplementary Figure 5.** Size separation of polydisperse hydrogel MSGs by sequential centrifugation. (**a**) Sequential centrifugation steps can be used to separate smaller hydrogel microspheres (scale bar = 50 μm), (**b**) as characterized by decreasing mean diameters, n = 100.


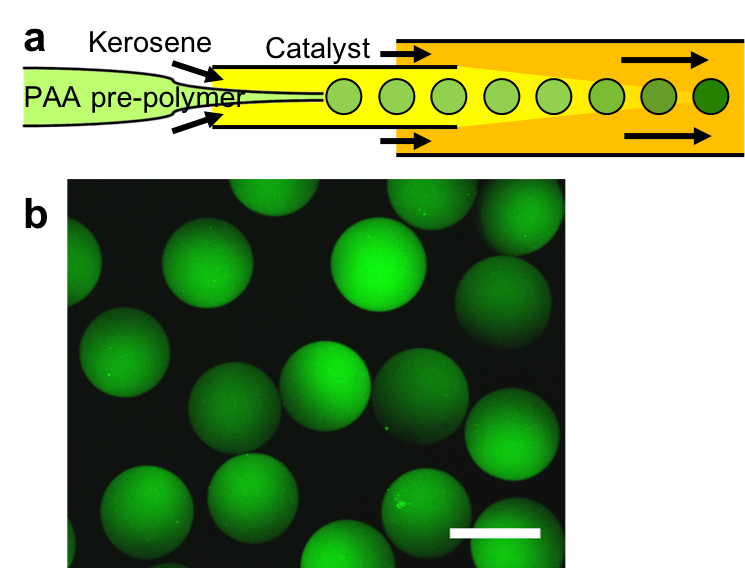


**Supplementary Figure 6.** Production of monodispersed hydrogel MSGs via microfluidic droplet generation. (**a**) A microfluidic droplet generator system consisting of a pulled circular micropipette inserted into a square glass tube was used to synthesize hydrogel microspheres of relatively uniform size. Droplets of polyacrylamide pre-polymer with fluorescein methacrylate monomers were generated in kerosene. A catalyst was added downstream of the droplet generation site, and hydrogel microspheres were allowed to polymerize. (**b**) The process enables the rapid production of a large number of uniform hydrogel microspheres (scale bar = 200 µm).


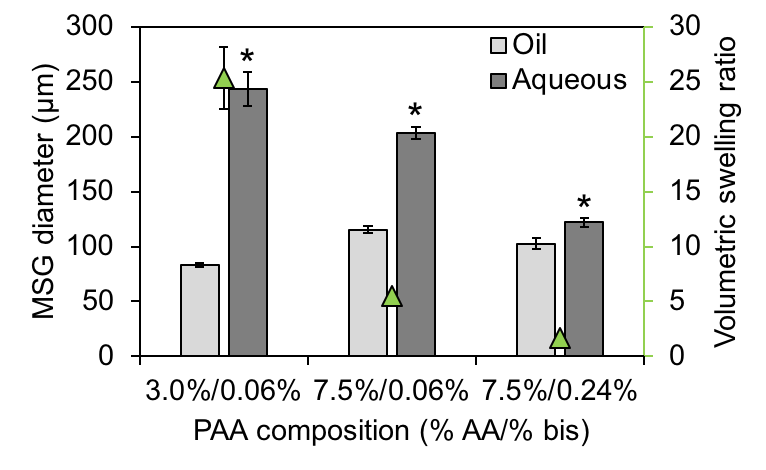


**Supplementary Figure 7.** Volumetric swelling ratio of hydrogel MSGs in oil and aqueous phases. The soft hydrogels developed here swell significantly in aqueous medium, making it challenging to fabricate very small MSGs of uniform size. Data reported as mean ± standard deviation, n > 20, * indicates p < 0.05 (one-way ANOVA with Tukey post-hoc pairwise comparisons).

**
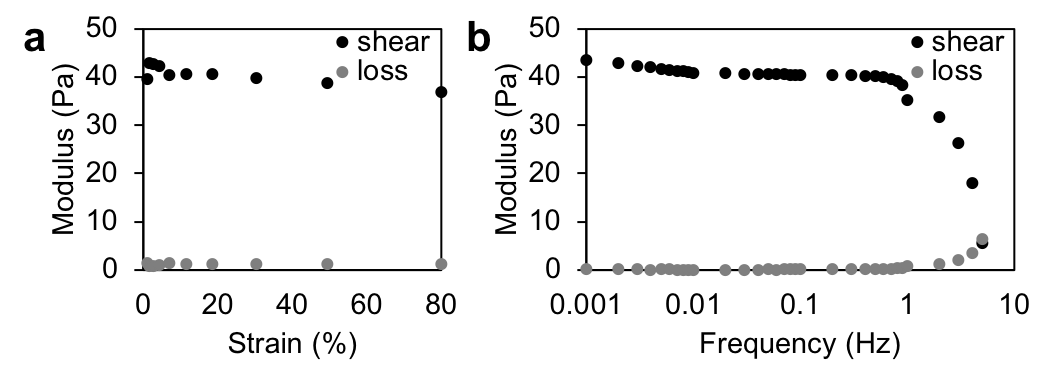
Supplementary Figure 8.** Shear rheometry characterization of bulk, fluorescently-labelled polyacrylamide hydrogels. These bulk measurements demonstrate linear elastic material properties with negligible loss modulus (**a**) over large strain range (data repeated in main manuscript Fig. 2a) and (**b**) for load frequencies less than 1 Hz. For frequencies greater than 1 Hz, the reduced shear modulus is likely due to slippage of the hydrogel on the rheometer plates. However, frequencies greater than 1 Hz are unlikely to be relevant to the current application of MSGs in multicellular spheroids.

**
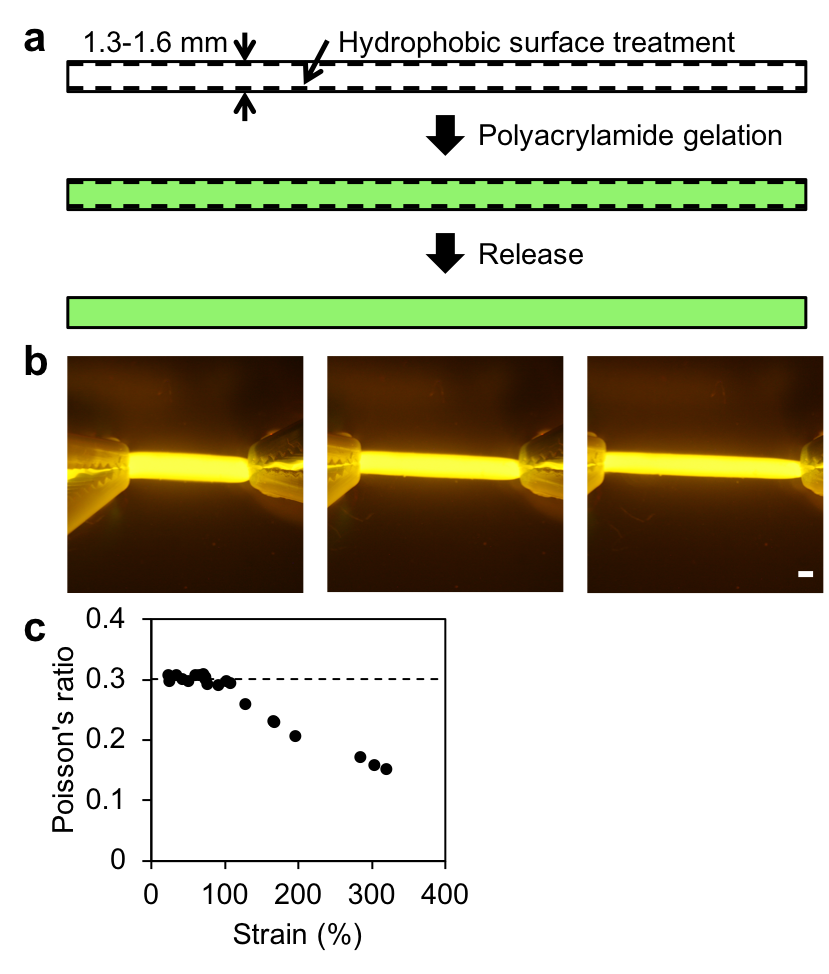
**

**Supplementary Figure 9.** Measurement of Poisson’s ratio of the fluorescently-labelled polyacrylamide hydrogel formulations used in this work. (**a**) Polyacrylamide hydrogel “strings” were fabricated within glass capillaries (internal diameter of 1.3-1.6 mm) that had been pre-treated to be hydrophobic. Following gelation, polyacrylamide hydrogel strings were released from the glass capillary and swelled for 24 hours before (**b**) stretching axially (scale bar = 1 mm) under a fluorescent dissecting microscope. The deformations in the transverse and axial directions were measured to compute the Poisson’s ratio which (**c**) remains constant for strains up to 120% (data repeated in main manuscript Fig. 2b).


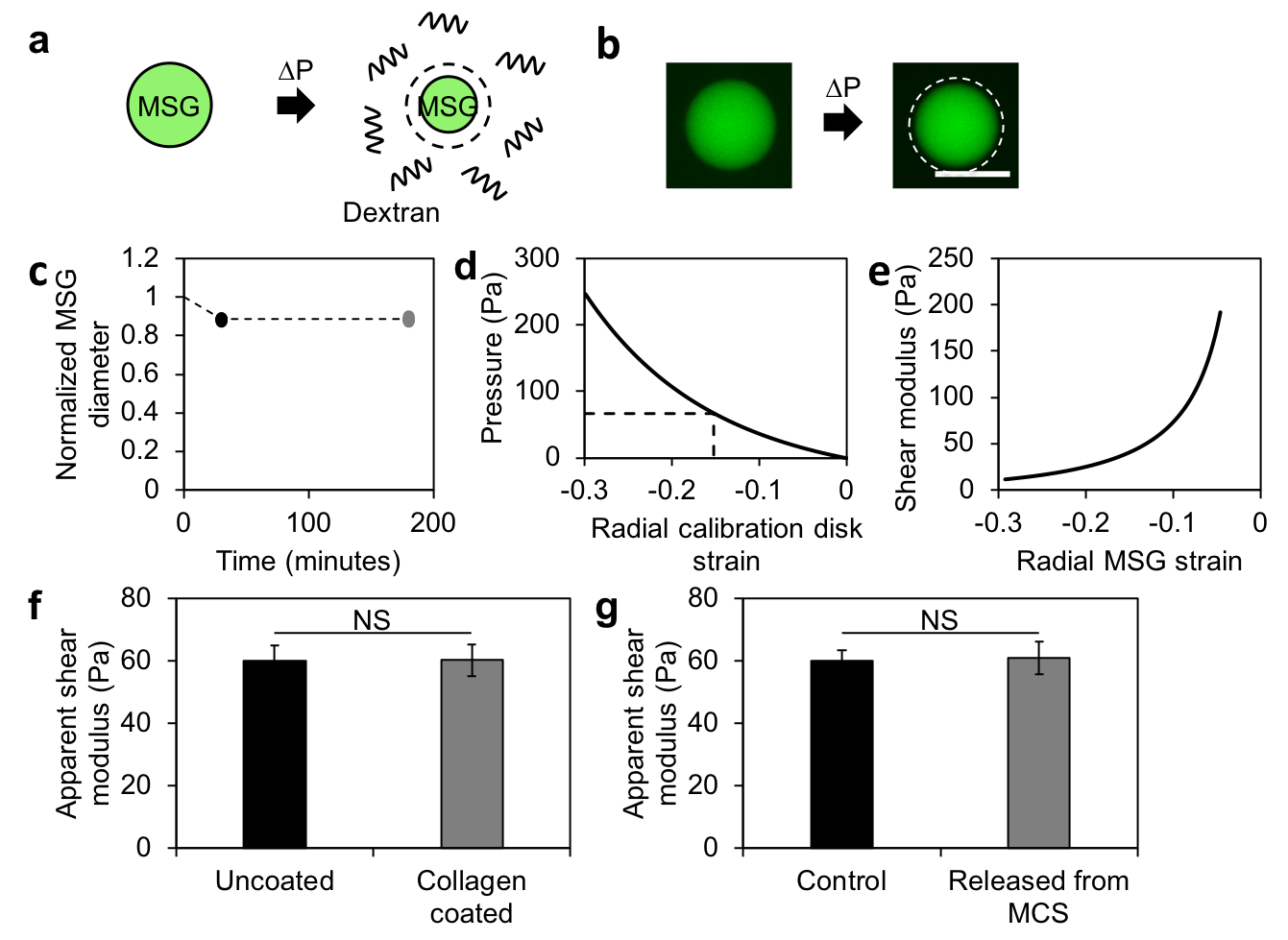


**Supplementary Figure 10.** Measurement of MSG mechanical properties by application of osmotic pressure. An aqueous solution of long-chain dextran (500 kDa) was used to exert an osmotic pressure on polyacrylamide hydrogels. The dextran polymer chains are too large to enter the polyacrylamide pores, and are therefore excluded from the hydrogel. The osmotic pressure differential forces water out of the MSG, which deforms in proportion to the MSG mechanical compliance. (**a**) A schematic representation and (**b**) fluorescent microscope images (scale bar = 50 μm) depicting hydrogel contraction when exposed to 100 mg/mL of dextran solution. (**c**) MSG sizes remain constant after 3 hours in the dextran solution, confirming that dextran chains are excluded from the polymer matrix (n = 19). The system was calibrated against osmotic pressure-induced deformation of a bulk disk-shaped polyacrylamide hydrogel sample (diameter = 13 mm) for which the shear modulus was established using conventional shear rheometry. (**d**) A finite element simulation was developed to determine the effective osmotic pressure generated by a 100 mg/mL solution of dextran. The parametric sweep of external pressures on samples was used to determine that 100 mg/mL of dextran exerts 67 Pa pressure on the hydrogel surface. Next, this osmotic pressure value was applied to (**e**) a parametric sweep of shear modulus in the isotropic compression of a spherical MSG. (**f**) Osmotic pressure measurements on MSGs indicates that collagen coating does not significantly alter mechanical rigidity of the MSG (n = 24, p = 0.782). (**g**) No significant differences were found between coated MSGs (control) and MSGs that had been removed from spheroids after two days of culture by detergent-based extraction (released), demonstrating that MSG properties remain constant even after embedding within the tissue of interest (n = 16-19, p = 0.837). All data reported as mean ± standard deviation. NS indicates no significant differences (one-way ANOVA with Tukey post-hoc pairwise comparisons).


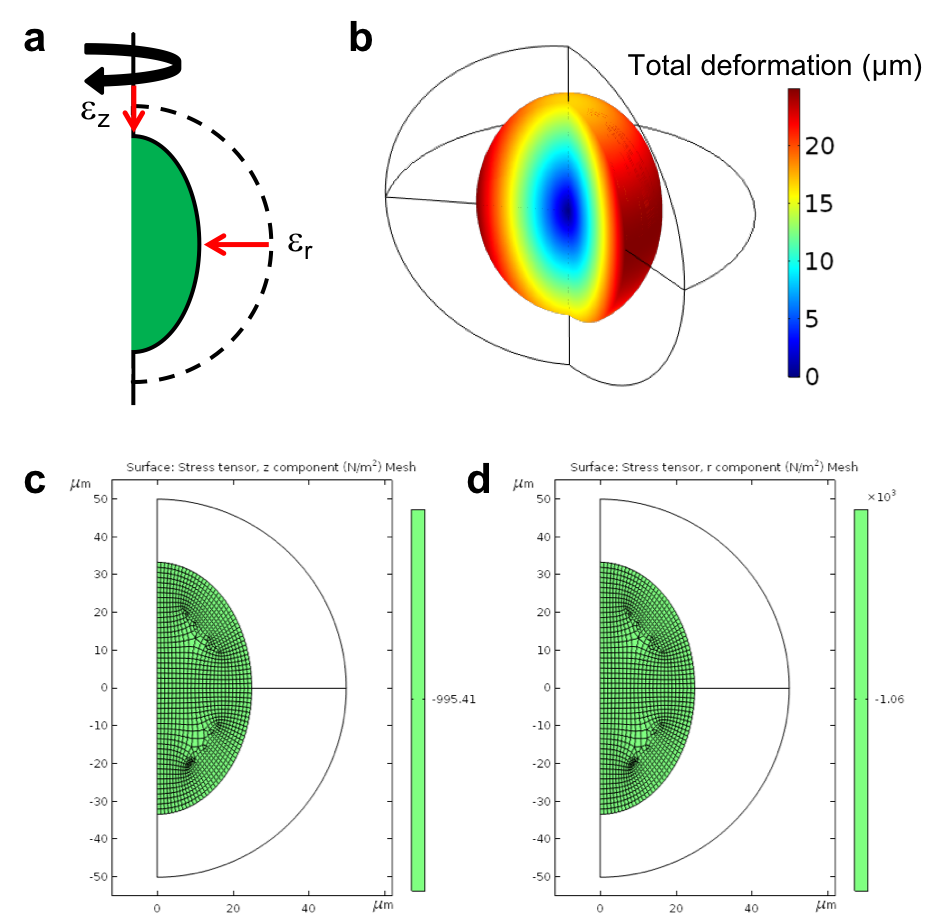


**Supplementary Figure 11.** Finite element model to simulate multiaxial MSG deformation. (**a**) Schematic of the two-dimensional axisymmetric model along with strain conditions applied throughout the bead domain. (**b**) Representative image of a partially revolved axisymmetric MSG bead deforming under -0.33 axial strain and -0.5 radial strain domain conditions. Corresponding (**c**) axial and (**d**) radial stresses are confirmed to be uniform throughout the MSG, consistent with the assumption of viscous flow in the surrounding tissue.

**
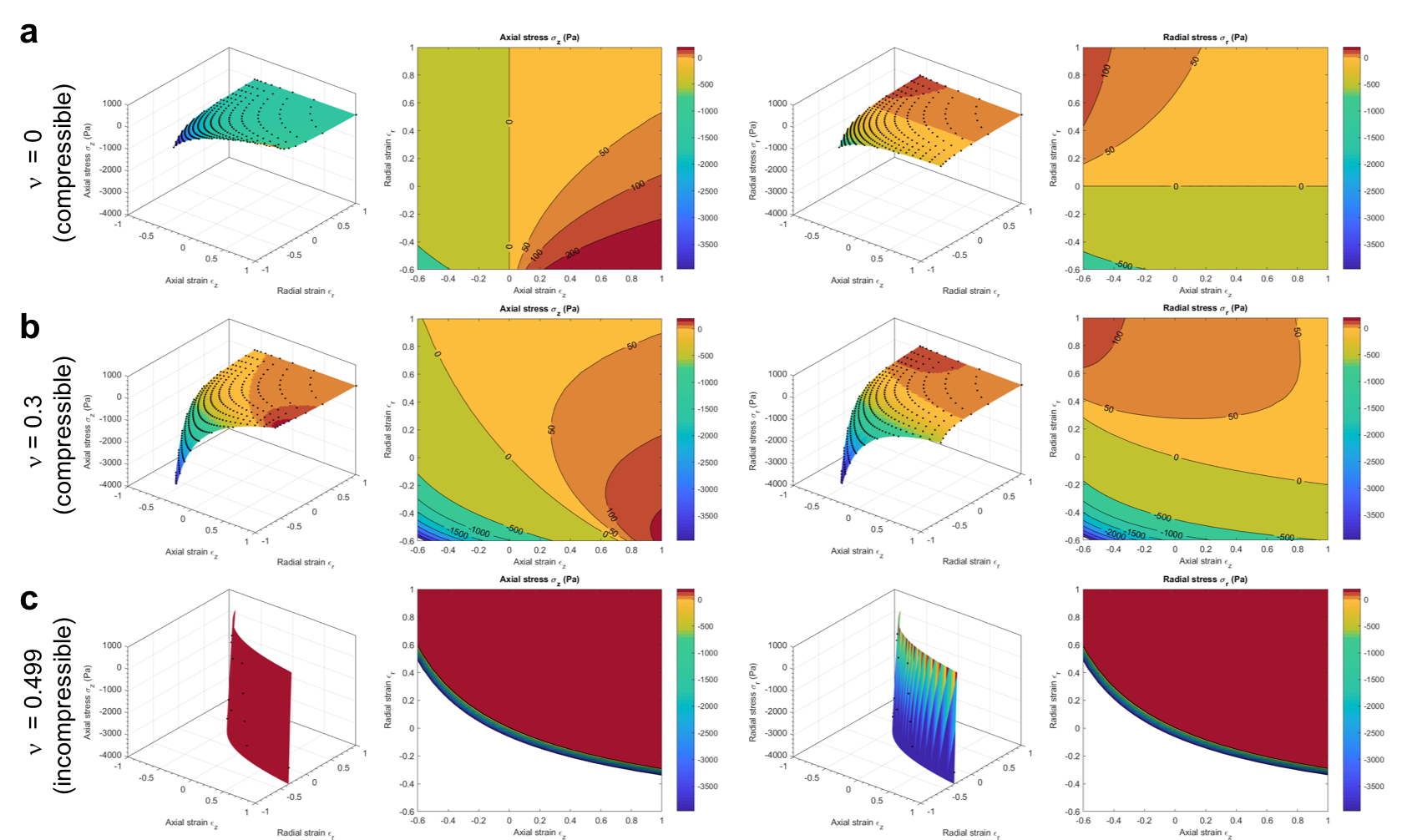
Supplementary Figure 12.** Compressibility of the hydrogel is an important parameter in generating unique solutions for axial and radial stresses based on measured MSG deformation. Finite element simulations relating axial (z) and radial (r) stresses with axial and radial strains for (**a**) perfectly compressible (ν = 0), (**b**) actual (ν = 0.3) and (**c**) incompressible (ν = 0.499) materials. For perfectly compressible materials, strains in the axial and radial directions are only weakly coupled to radial and axial stresses respectively. On the other end of the spectrum, as the material approaches (**c**) incompressibility (ν = 0.499), microsphere deformations cannot be resolved into unique combinations of axial and radial stress. Hence, the use of compressible materials enables the measurement of both isotropic and anisotropic stress components in the system.

**
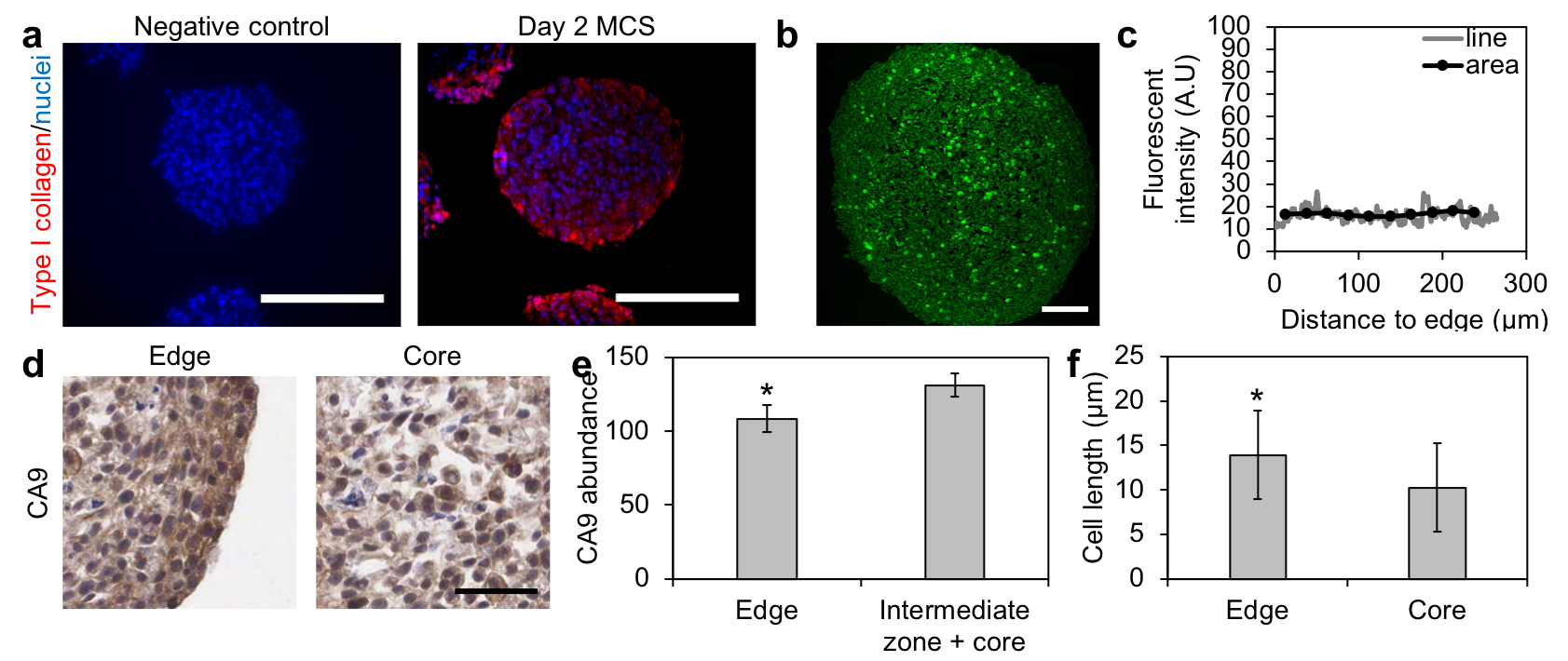
**

**Supplementary Figure 13.** Characterization of MCS structure 48 hours after formation. (**a**) Type I collagen fluorescent immunostaining on sectioned MCS confirms that HS-5 fibroblasts secrete Type I collagen over 2 days of culture (scale bar = 250 μm). Negative control performed without primary antibody confirms that the signal detected is not a result of non-specific binding. (**b, c**) Second harmonic imaging of collagen shows no spatial variation in ECM organization within spheroid sections. (**b**) Second harmonic imaging of mature collagen on sectioned spheroids indicates no spatial variations (scale bar = 100 μm). (**c**) Quantification of fluorescence intensity along a line segment and normalized to quantified area shows no variation in mature collagen content within MCS. (**d, e**) The core of spheroid cultures does not exhibit hypoxia as indicated by immunohistochemical analysis for carbonic anhydrase 9 (CA9), a marker of hypoxia. (**d**) A representative immunohistochemical section is shown (scale bar = 50 μm). (**e**) Immunohistochemical analysis shows similar abundance of CA9 in cells located in the periphery and the core of the spheroid, indicating an absence of an oxygen gradient. Data reported as mean ± standard deviation, n = 13, * indicates p < 0.001 (one-way ANOVA with Tukey post-hoc pairwise comparisons). (f) Characterization of cell elongation in H&E stained spheroid sections. Data reported as mean ± standard deviation, n = 30 over 3 spheroids, * indicates p < 0.001 (one-way ANOVA with Tukey post-hoc pairwise comparisons).

**
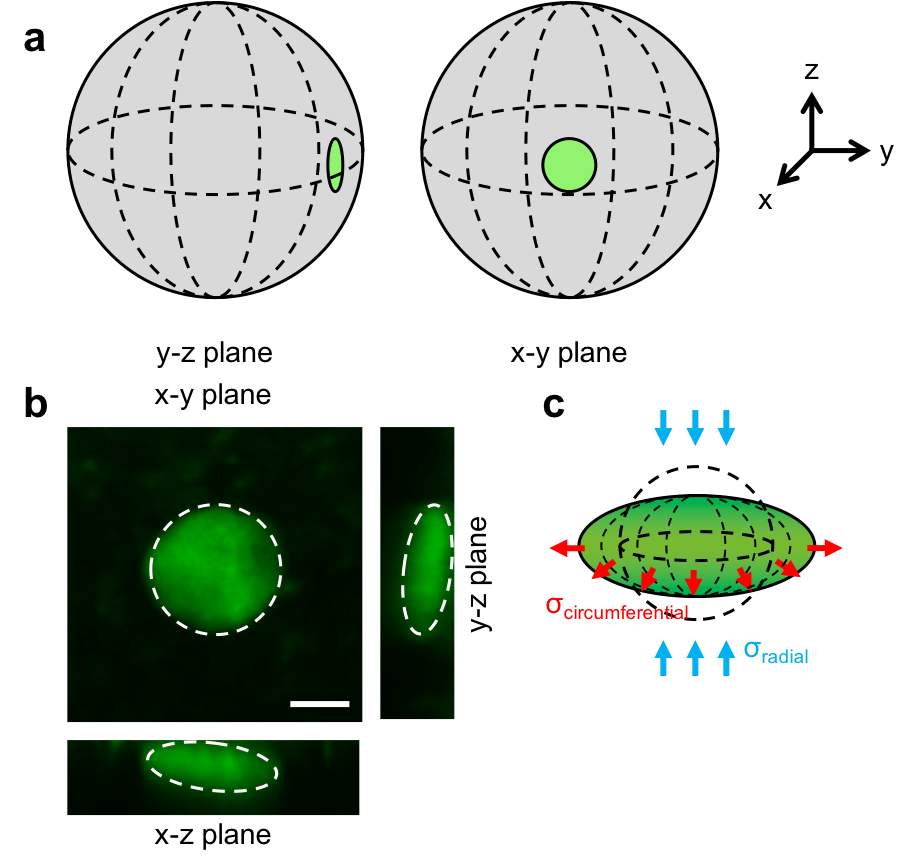
**

**Supplementary Figure 14.** Reconstructed confocal images of MSGs embedded at the periphery of the MSG confirms symmetric deformaton of the MSGs within spheroid cultures. (a) Schematic representation of the MSG location within the spheroids, and (b) reconstructed confocal images of MSGs close to the surface show deformations as expected based on spherical symmetry (scale bar = 25 μm), (c) The ‘pancake’-like morphologies adopted demonstrate two main axes of uniform deformation (radial and circumferential), arising from compressive radial stress and tensional circumferential stress.

**
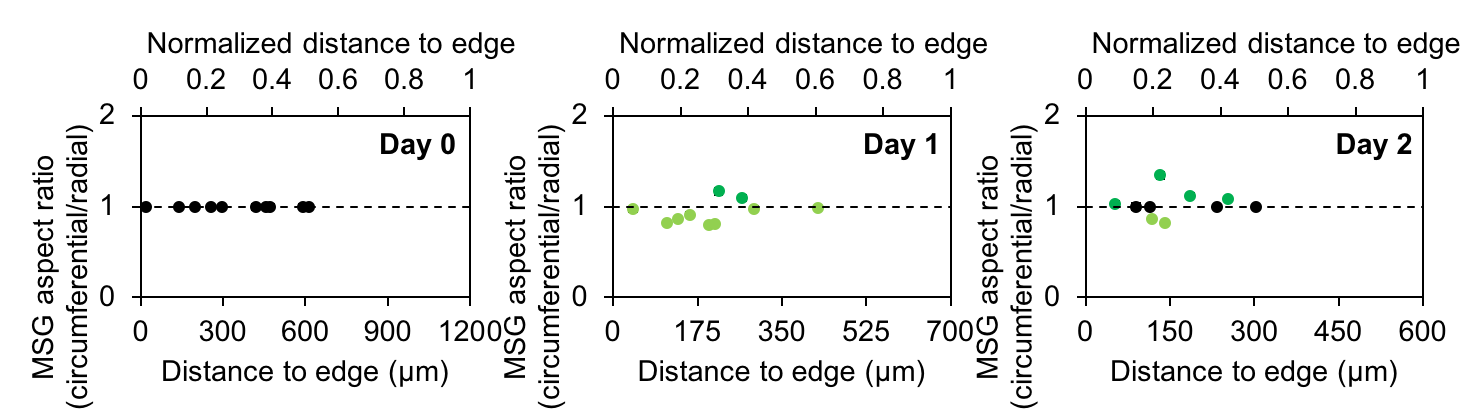
**

**Supplementary Figure 15.** Measurement of circumferential and radial microsphere strains indicate some spatial pattern in microsphere orientation within blebbistatin treated MCS in the first 24 hours. This preferential orientation is lost by day 2 in blebbistatin treated MCS cultures. Data reported as mean ± standard deviation for measurement error.


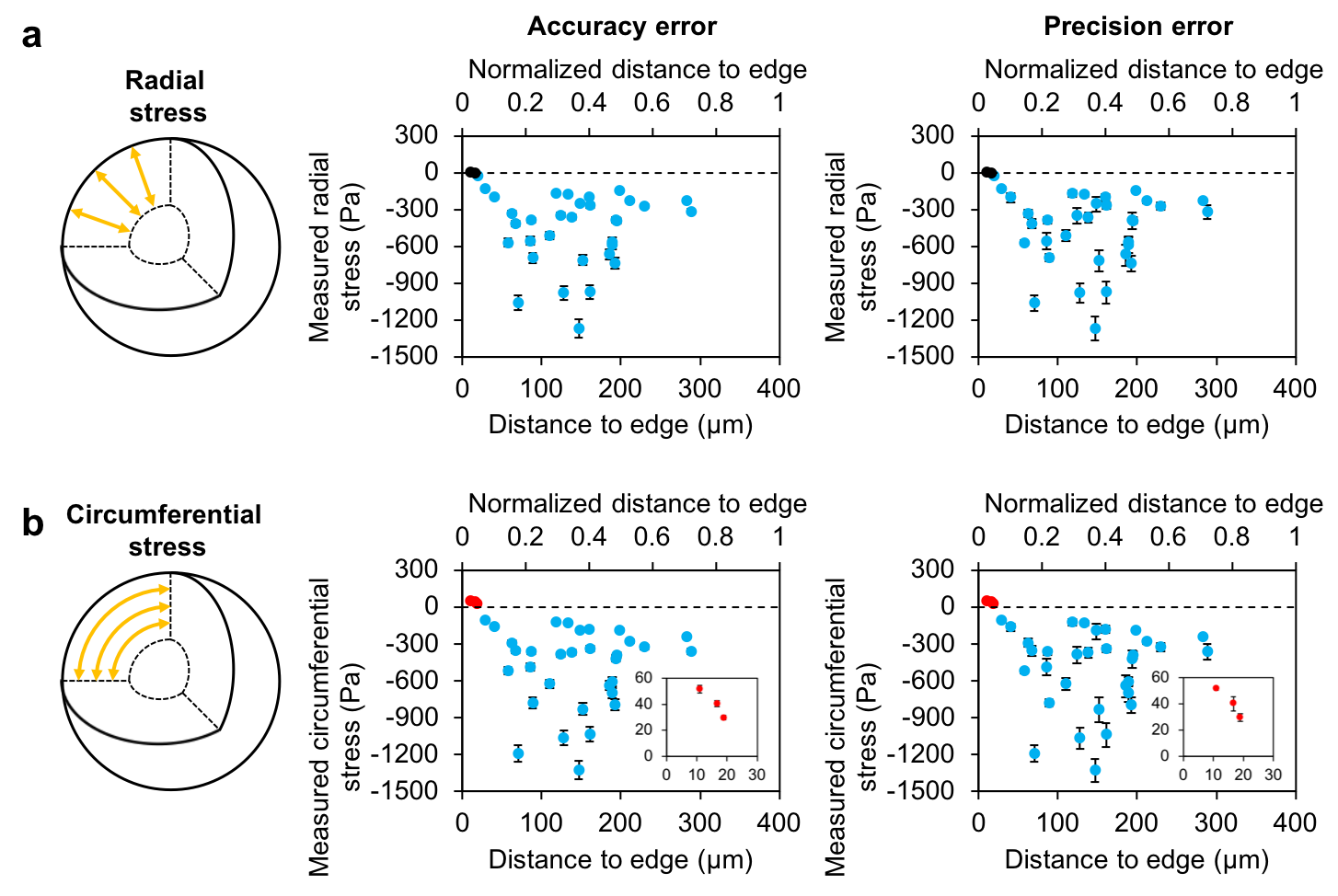


**Supplementary Figure 16.** Comparison of MSG errors associated with uncertainties in MSG modulus (accuracy) and strain measurement error (precision) in the (**a**) radial and (**b**) circumferential directions at day 2 of culture. Red data points represent tensional stress measurements, blue data points represent compressional stress measurements, and black data points represent stress measurements close to zero (-10 Pa to +10 Pa). Insets depict closer view of measured tensional stresses. Accuracy errors correspond to errors of 6% in stress readings, while precision errors were generated based on Monte Carlo simulations of error assuming a Gaussian normal distribution of values for repeated measurements of radial and circumferential bead dimensions. Both errors are combined and reported in the main manuscript figures (Fig. 4).


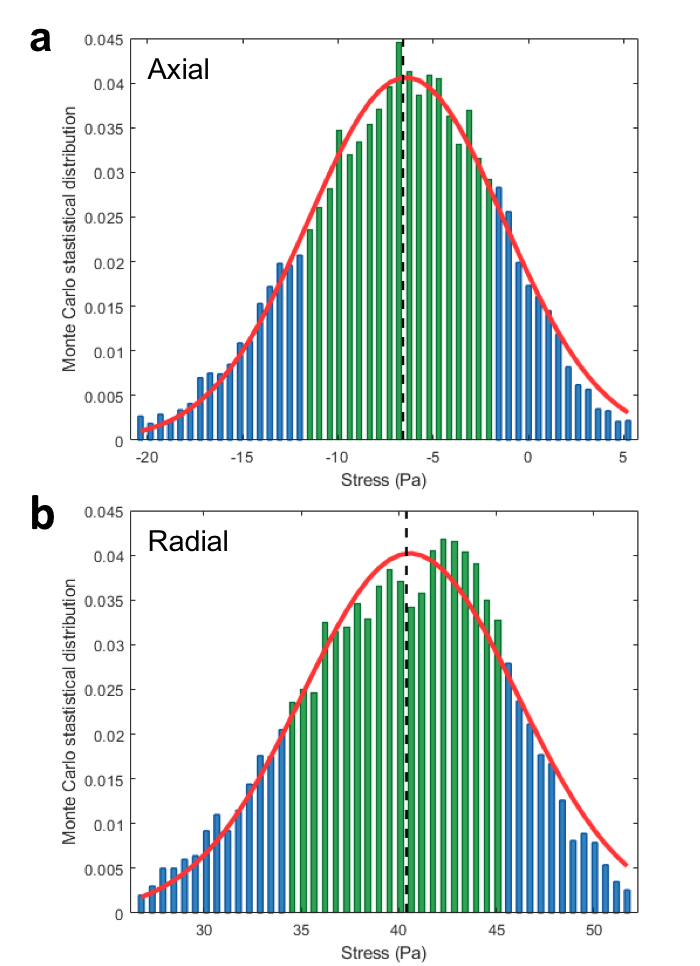


**Supplementary Figure 17.** Representative bar graphs of Monte Carlo estimates in stress measurement uncertainties arising from errors in measurement of MSG deformation. Repeated measurement of MSG dimensions was used to estimate the error in analysis of MSG size along the axial and radial axes. Assuming a Gaussian normal distribution of measurements in both the radial and circumferential axes for each data point, 10,000 randomly generated deformation values were converted to stresses through the non-linear interpolation function described in Fig. 2e, f. (**a, b**) Representative datasets from (**a**) axial and (**b**) radial stress Monte Carlo statistical distributions for a single axial compression-radial tension MSG data point (-6.50 Pa in the axial direction; +40.46 Pa in the radial direction). Mean stress values (dashed line) and their respective 95% confidence intervals (green section) are obtained empirically from the randomly generated dataset around each point. Similar curves were generated for every datapoint analyzed, and the 95% confidence intervals for each point are plotted as estimates of error in Supplementary Figure 16. These errors are then combined with errors in systemic accuracy to determine the total measurement error, values reported in Fig. 4, and in supplemental tables.


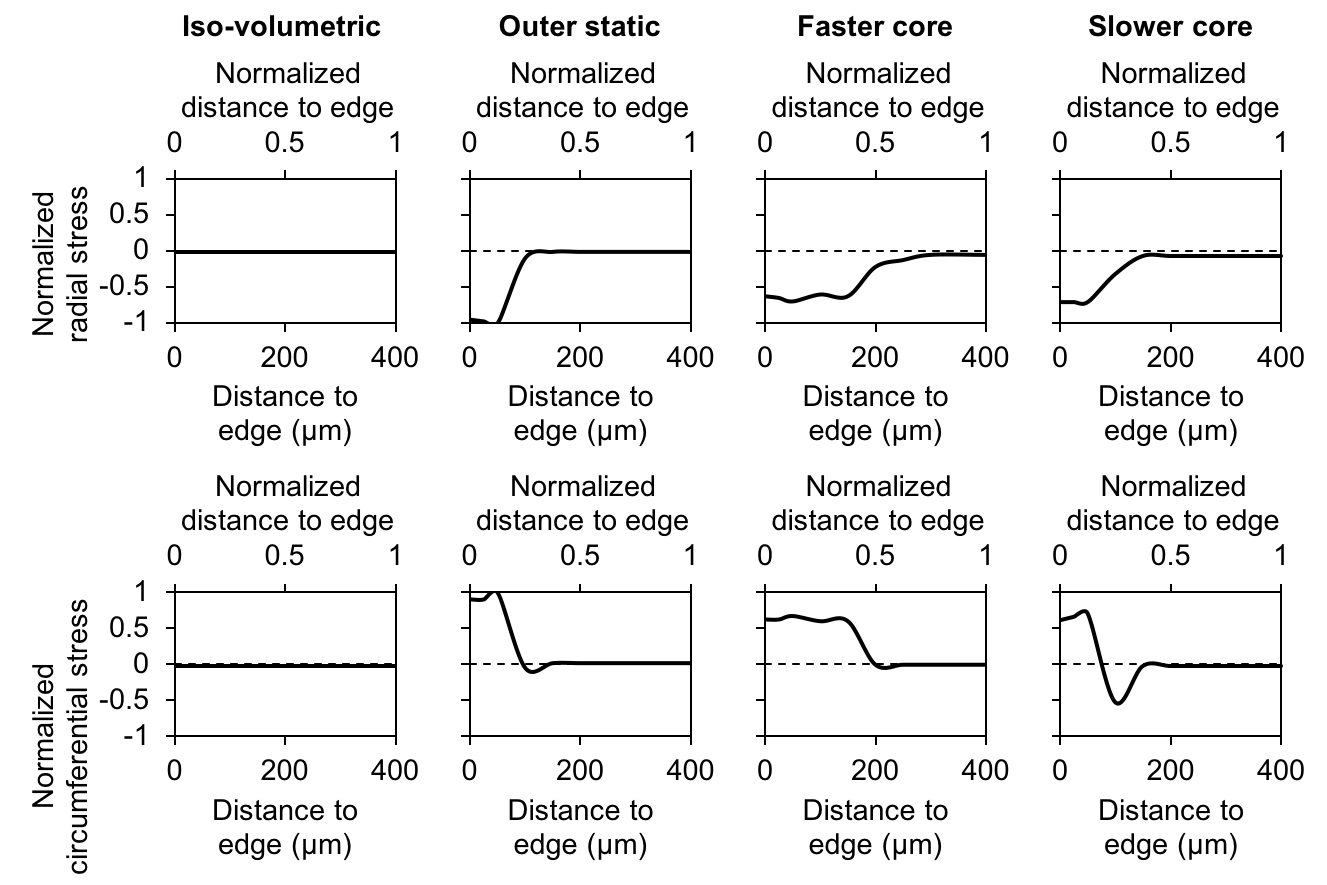


**Supplementary Figure 18.** Finite element model to simulate cell-generated mechanical stresses within MCS cultures driven by differences in cell proliferation. Finite element simulations show internal stress profiles generated with (1) iso-volumetric growth in the edge, intermediate zone, and core regions; (2) a non-proliferative edge with the intermediate zone and core growing at the same rate; (3) a non-proliferative edge with the core growing faster than the intermediate zone; and (4) a non-proliferative edge with the core growing slower than the intermediate zone. The stress profiles indicate that a non-proliferating edge is required to obtain a shell of tension in the circumferential direction around the outer layer of the MCS, as the static edge confines the growing internal layers of the spheroid, much like the walls of an inflating balloon. Mismatches in growth between the intermediate zone and core generate different stress profiles, where a peak in compression can only be obtained when the core is growing at a slower rate than the intermediate zone.


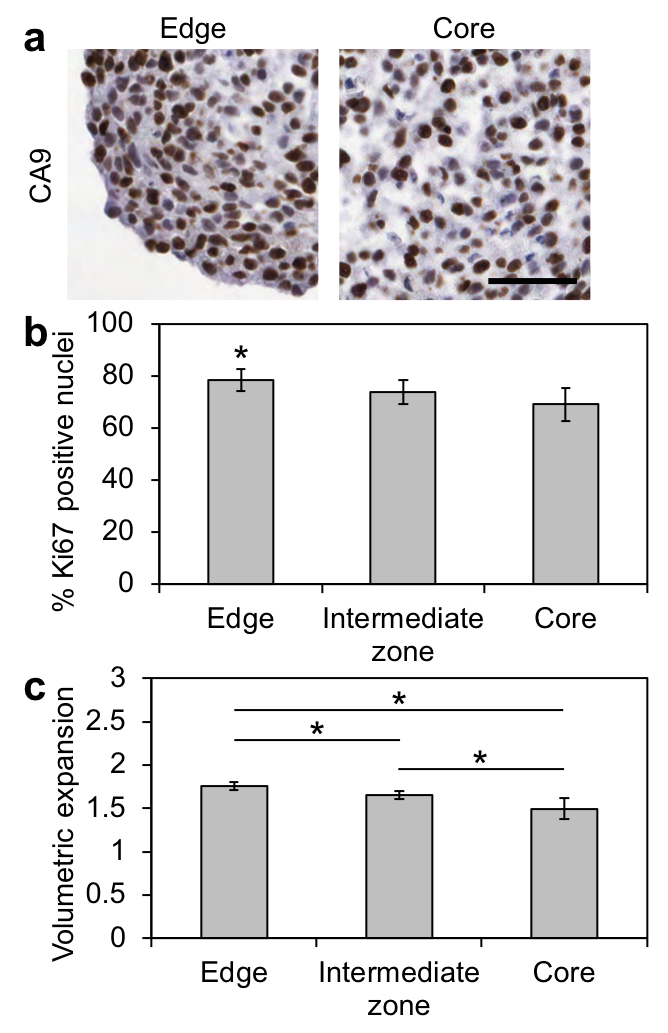


**Supplementary Figure 19.** The intermediate zone of the spheroid does not exhibit maximal cell proliferate rate by immunohistochemical analysis of Ki67, a marker of cell proliferation. (**a**) A representative immunohistochemical section is shown (scale bar = 50 μm). (**b**) Immunohistochemical analysis shows significantly higher percentage of cells positive for nuclear Ki67 in the periphery compared to the core of the spheroid, suggesting an increased rate of proliferation towards the edge of the spheroid (scale bar = 50 μm). Data reported as mean ± standard deviation, n = 11, * indicates p < 0.05 when compared to other spatial locations (one-way ANOVA with Tukey post-hoc pairwise comparisons). (**c**) Volumetric expansion after one cycle of cell division calculated based on nuclei packing and Ki67 staining results. Data reported as mean ± standard deviation, n = 9, * indicates p < 0.05 (one-way ANOVA with Tukey post-hoc pairwise comparisons).

**
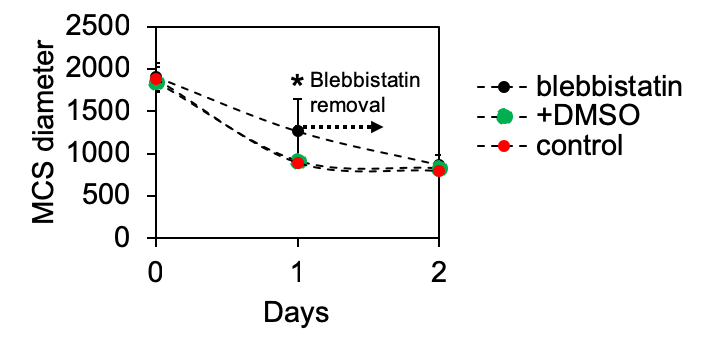
**

**Supplementary Figure 20**. Confirmation of blebbistatin activity through quantification of spheroid size. Compaction is significantly reduced in MCS when actomyosin contractility is inhibited with blebbistatin (day 1), but is regained when the drug is washed out (day 2). Data reported as mean ± standard deviation, n = 11, * indicates p < 0.01 (Student’s t-test).

**Supplementary Table 1.** Radial and circumferential strains for control data set.

|  | Distance to edge (μm) | Radial strain | Circumferential strain | Radial strain standard deviation | Circumferential strain standard deviation |
| --- | --- | --- | --- | --- | --- |
| Day 0 | 253.271667 | -0.0173117 | -0.0173117 | 0.00750415 | 0.00750415 |
|  | 125.839 | 0.03100127 | 0.03100127 | 0.01065199 | 0.01065199 |
|  | 22.1433333 | -0.0185852 | -0.0185852 | 0.0095284 | 0.0095284 |
|  | 80.5896667 | 0.0153115 | 0.0153115 | 0.00577389 | 0.00577389 |
|  | 250.139 | -0.0434611 | -0.0434611 | 0.0090087 | 0.0090087 |
|  | 72.9496667 | -0.0146369 | -0.0146369 | 0.01624392 | 0.01624392 |
|  | 78.2076667 | -0.023329 | -0.023329 | 0.01774364 | 0.01774364 |
|  | 533.014333 | -0.0119751 | -0.0119751 | 0.01218096 | 0.01218096 |
|  | 79.9646667 | -0.0024966 | -0.0024966 | 0.00878492 | 0.00878492 |
| Day 1 | 264.881667 | -0.0756392 | -0.3650069 | 0.01475975 | 0.02080695 |
|  | 201.624667 | -0.4435282 | -0.2124585 | 0.01250441 | 0.0142695 |
|  | 102.540667 | -0.1189901 | -0.4605942 | 0.00701969 | 0.01079038 |
|  | 192.022333 | -0.2914491 | -0.4571806 | 0.01583851 | 0.01248298 |
|  | 122.896667 | -0.4644802 | -0.1467778 | 0.0301295 | 0.00860798 |
|  | 142.887333 | -0.4953367 | -0.0065193 | 0.00939172 | 0.00809539 |
|  | 215.912333 | -0.0337602 | -0.3270834 | 0.001718 | 0.01150695 |
|  | 112.064 | -0.1931875 | -0.3890554 | 0.0206755 | 0.00389932 |
|  | 163.003667 | -0.1452642 | -0.4215483 | 0.01621957 | 0.0095584 |
|  | 146.594333 | -0.177023 | -0.4728582 | 0.02426695 | 0.00460169 |
|  | 140.748 | -0.1308068 | -0.4913635 | 0.02469393 | 0.01827656 |
|  | 142.519 | -0.2168688 | -0.3912013 | 0.01278379 | 0.02710338 |
|  | 134.803333 | -0.3127343 | -0.2784181 | 0.00849512 | 0.01562575 |
|  | 155.406 | -0.5408411 | -0.38305 | 0.00354685 | 0.02167709 |
|  | 18.788 | -0.1664079 | 0.12768318 | 0.0128192 | 0.02533022 |
|  | 293.869333 | -0.2016779 | -0.2935707 | 0.0148574 | 0.01092408 |
|  | 319.972 | -0.3827587 | -0.1639899 | 0.00870317 | 0.01494147 |
| Day 2 | 188.8285 | -0.2580223 | -0.4048553 | 0.02098765 | 0.0081292 |
|  | 195.138667 | -0.3040303 | -0.3072777 | 0.01295648 | 0.01260982 |
|  | 88.716 | -0.2227406 | -0.4594171 | 0.01186624 | 0.00509735 |
|  | 146.8565 | -0.3639815 | -0.4997769 | 0.02346986 | 0.00832615 |
|  | 70.0875 | -0.2281152 | -0.5271602 | 0.01903957 | 0.00670429 |
|  | 124.007 | -0.2009152 | -0.3465628 | 0.03298533 | 0.02770151 |
|  | 86.7036667 | -0.3705685 | -0.2628322 | 0.0162228 | 0.00719932 |
|  | 57.3656667 | -0.4689408 | -0.2761605 | 0.00528345 | 0.00596835 |
|  | 138.430333 | -0.2861065 | -0.3040828 | 0.00647076 | 0.02165226 |
|  | 40.2276667 | -0.3510402 | -0.1272162 | 0.04559503 | 0.01905851 |
|  | 10.9856667 | -0.2148388 | 0.31090689 | 0.0158255 | 0.00763435 |
|  | 85.6476667 | -0.504119 | -0.2404744 | 0.01875568 | 0.02455746 |
|  | 67.1053333 | -0.4622995 | -0.2061528 | 0.00837447 | 0.02187356 |
|  | 160.014333 | -0.2967276 | -0.1737487 | 0.02129463 | 0.02410194 |
|  | 29.206 | -0.2486057 | -0.1164766 | 0.01321434 | 0.01303668 |
|  | 282.744 | -0.1935859 | -0.2687895 | 0.01394602 | 0.00398711 |
|  | 288.575667 | -0.1706106 | -0.3447979 | 0.02672242 | 0.02790839 |
|  | 127.933667 | -0.2815952 | -0.4925533 | 0.00332189 | 0.0138056 |
|  | 109.956333 | -0.1099068 | -0.4578969 | 0.0217457 | 0.01095201 |
|  | 192.460667 | -0.2794271 | -0.4438034 | 0.02203337 | 0.01126994 |
|  | 148.156333 | -0.4242247 | -0.1161216 | 0.02569421 | 0.05658027 |
|  | 189.080333 | -0.1426186 | -0.4670056 | 0.0267774 | 0.01341946 |
|  | 62.473 | -0.3967844 | -0.2068374 | 0.01197061 | 0.01966358 |
|  | 151.817667 | -0.1729637 | -0.4856882 | 0.02108204 | 0.01870056 |
|  | 160.749333 | -0.3158537 | -0.4755891 | 0.00907175 | 0.01540138 |
|  | 193.553667 | -0.2383188 | -0.3461599 | 0.01847675 | 0.0309486 |
|  | 198.174 | -0.0764169 | -0.2676159 | 0.02499724 | 0.00517769 |
|  | 160.980333 | -0.0868809 | -0.3623651 | 0.01908261 | 0.01223852 |
|  | 118.081667 | -0.3606838 | -0.0797439 | 0.02692123 | 0.02829732 |
|  | 133.631667 | -0.3666899 | -0.087223 | 0.01032526 | 0.02177059 |
|  | 18.941 | -0.2678937 | 0.20217184 | 0.03220038 | 0.00766611 |
|  | 185.406 | -0.4363358 | -0.3340389 | 0.0146086 | 0.02669782 |
|  | 16.6946667 | -0.2225128 | 0.24081644 | 0.01776937 | 0.02971592 |
|  | 229.6955 | -0.1318971 | -0.3409722 | 0.01519917 | 0.01715384 |
|  | 211.6955 | -0.1148664 | -0.3214346 | 0.01007977 | 0.01006305 |

**Supplementary Table 2.** Radial and circumferential stresses for control data set.

|  | Distance to edge (um) | Radial stress (Pa) | Circumferential stress (Pa) | (-) Radial stress standard deviation (Pa) | (+) Radial stress standard deviation (Pa) | (-) Circumferential stress standard deviation (Pa) | (+) Circumferential stress standard deviation (Pa) |
| --- | --- | --- | --- | --- | --- | --- | --- |
| Day 0 | 253.271667 | -7.301111 | -7.46598 | 2.673557 | 2.593557 | 3.083422 | 3.04337 |
|  | 125.839 | 10.867287 | 10.744592 | 3.087121 | 2.934217 | 3.466865 | 3.361118 |
|  | 22.1433333 | -7.862515 | -7.982986 | 3.387298 | 3.303862 | 3.817699 | 3.748761 |
|  | 80.5896667 | 5.485827 | 5.396788 | 1.772409 | 1.794597 | 1.9167 | 1.947585 |
|  | 250.139 | -19.706897 | -19.664205 | 4.412946 | 4.344097 | 4.804758 | 4.486302 |
|  | 72.9496667 | -6.208612 | -6.239468 | 5.412413 | 5.225729 | 6.12386 | 5.721876 |
|  | 78.2076667 | -9.84552 | -10.047527 | 6.671968 | 5.801549 | 7.252188 | 6.693407 |
|  | 533.014333 | -5.048624 | -5.117344 | 3.832863 | 3.825252 | 4.514839 | 4.266862 |
|  | 79.9646667 | -1.076698 | -1.120348 | 2.643903 | 2.435971 | 3.093488 | 2.799602 |
| Day 1 | 264.881667 | -261.75762 | -334.43597 | 38.035727 | 52.732491 | 47.45485 | 64.940733 |
|  | 201.624667 | -398.18737 | -344.16315 | 30.991038 | 53.707327 | 51.267018 | 50.46976 |
|  | 102.540667 | -531.06431 | -644.70631 | 37.356059 | 68.0837 | 65.569626 | 81.289143 |
|  | 192.022333 | -819.67546 | -879.47562 | 69.837459 | 117.734797 | 111.592762 | 125.020398 |
|  | 122.896667 | -327.89236 | -260.0256 | 41.668457 | 60.388879 | 54.455746 | 47.888101 |
|  | 142.887333 | -216.22689 | -127.97752 | 11.201104 | 23.744437 | 23.19474 | 17.009063 |
|  | 215.912333 | -179.82457 | -245.99289 | 13.225495 | 23.783057 | 24.064874 | 32.007787 |
|  | 112.064 | -429.16753 | -484.49093 | 27.561167 | 52.50293 | 51.578255 | 51.567937 |
|  | 163.003667 | -450.84812 | -534.5096 | 33.832836 | 59.855218 | 56.470742 | 66.421246 |
|  | 146.594333 | -667.01141 | -772.09647 | 47.534088 | 86.904745 | 83.335314 | 87.493245 |
|  | 140.748 | -663.59051 | -796.52014 | 93.714852 | 132.293223 | 119.533007 | 152.127711 |
|  | 142.519 | -465.21639 | -515.17424 | 75.8833 | 103.687071 | 94.450046 | 118.301684 |
|  | 134.803333 | -347.38045 | -339.36633 | 29.590402 | 49.473281 | 45.958238 | 51.596852 |
|  | 155.406 | -1171.3065 | -1112.9573 | 125.031734 | 193.401521 | 180.029051 | 194.988333 |
|  | 18.788 | -12.45476 | 20.202178 | 5.393404 | 5.971468 | 5.493387 | 7.784411 |
|  | 293.869333 | -263.00106 | -283.75706 | 20.521845 | 35.563459 | 34.304281 | 37.504438 |
|  | 319.972 | -261.90548 | -217.68424 | 19.624759 | 34.776661 | 32.433646 | 33.587842 |
| Day 2 | 188.8285 | -561.74726 | -606.59078 | 74.345071 | 72.994292 | 74.597857 | 73.415059 |
|  | 195.138667 | -389.21268 | -390.04614 | 51.764252 | 50.066928 | 53.518171 | 51.114409 |
|  | 88.716 | -692.93029 | -775.17568 | 70.596834 | 69.651415 | 75.706811 | 73.828534 |
|  | 146.8565 | -1265.8421 | -1324.417 | 176.092648 | 168.148629 | 175.724862 | 168.794673 |
|  | 70.0875 | -1058.3039 | -1187.6877 | 129.106966 | 124.482019 | 136.100324 | 131.188514 |
|  | 124.007 | -346.58811 | -383.7132 | 88.498538 | 75.11601 | 95.310407 | 80.613553 |
|  | 86.7036667 | -388.38562 | -362.7006 | 47.290177 | 45.288914 | 43.406404 | 41.605168 |
|  | 57.3656667 | -569.80526 | -517.59844 | 50.362442 | 50.991774 | 47.374011 | 47.727092 |
|  | 138.430333 | -363.20447 | -367.80412 | 63.61905 | 59.24869 | 69.366227 | 64.504504 |
|  | 40.2276667 | -198.99379 | -158.16559 | 53.440548 | 45.903549 | 42.860863 | 37.126817 |
|  | 10.9856667 | 2.728722 | 51.664435 | 2.758705 | 2.605397 | 4.11539 | 4.110623 |
|  | 85.6476667 | -554.0227 | -484.98441 | 104.268008 | 95.329613 | 99.417808 | 92.712085 |
|  | 67.1053333 | -413.91522 | -353.47635 | 65.203254 | 59.960379 | 64.104445 | 58.100034 |
|  | 160.014333 | -200.96727 | -177.51766 | 40.941258 | 37.310014 | 40.547429 | 37.110482 |
|  | 29.206 | -126.87073 | -104.83445 | 18.090161 | 17.607321 | 17.131145 | 16.499581 |
|  | 282.744 | -224.40981 | -240.62926 | 24.851578 | 23.482379 | 23.773275 | 22.704362 |
|  | 288.575667 | -314.27931 | -357.63049 | 78.688965 | 68.147019 | 88.249107 | 76.277875 |
|  | 127.933667 | -978.05519 | -1061.7975 | 138.80166 | 131.97834 | 152.938279 | 145.161453 |
|  | 109.956333 | -510.53929 | -624.53872 | 76.202414 | 73.624658 | 85.597569 | 81.837102 |
|  | 192.460667 | -736.68699 | -793.52568 | 111.211723 | 105.124773 | 114.388202 | 109.361181 |
|  | 148.156333 | -249.70145 | -189.79308 | 84.616506 | 69.884388 | 84.508178 | 69.713043 |
|  | 189.080333 | -587.96949 | -699.56491 | 100.321726 | 94.225167 | 109.655728 | 104.467225 |
|  | 62.473 | -331.50159 | -289.57881 | 52.55099 | 49.866248 | 51.475102 | 48.783613 |
|  | 151.817667 | -712.22459 | -827.89834 | 135.846737 | 122.513602 | 152.064473 | 139.094313 |
|  | 160.749333 | -970.83425 | -1032.3334 | 150.399661 | 140.79986 | 162.008984 | 151.48817 |
|  | 193.553667 | -387.43063 | -415.59427 | 93.288523 | 81.157794 | 103.267326 | 90.685279 |
|  | 198.174 | -145.45285 | -184.15277 | 24.225872 | 22.711096 | 22.790903 | 21.894121 |
|  | 160.980333 | -268.25797 | -337.4293 | 41.56183 | 41.131887 | 47.77239 | 46.948338 |
|  | 118.081667 | -167.9813 | -119.41575 | 38.930277 | 35.06345 | 35.279741 | 31.066598 |
|  | 133.631667 | -177.73477 | -128.68484 | 29.776894 | 27.4939 | 28.42619 | 26.117421 |
|  | 18.941 | -21.819972 | 29.703175 | 9.180939 | 8.412283 | 4.856412 | 4.423935 |
|  | 185.406 | -664.25138 | -633.86438 | 132.773095 | 119.351704 | 136.021535 | 120.612126 |
|  | 16.6946667 | -6.489743 | 40.463765 | 5.828272 | 5.302946 | 8.2697 | 7.276722 |
|  | 229.6955 | -272.29484 | -322.97951 | 46.735394 | 43.332894 | 54.426264 | 50.12556 |
|  | 211.6955 | -230.56426 | -278.4297 | 29.40033 | 28.336532 | 34.33804 | 33.707544 |

**Supplementary Table 3.** Radial and circumferential strains for blebbistatin data set.

|  | Distance to edge (μm) | Radial strain | Circumferential strain | Radial strain standard deviation | Circumferential strain standard deviation |
| --- | --- | --- | --- | --- | --- |
| Day 0 | 589.337333 | -0.0036178 | -0.0036178 | 0.01918289 | 0.01918289 |
|  | 138.919 | -0.0025079 | -0.0025079 | 0.01554925 | 0.01554925 |
|  | 256.589333 | -0.021659 | -0.021659 | 0.00523918 | 0.00523918 |
|  | 470.431667 | -0.0119578 | -0.0119578 | 0.01186768 | 0.01186768 |
|  | 418.662 | -0.0001081 | -0.0001081 | 0.02322107 | 0.02322107 |
|  | 611.276 | -0.0156326 | -0.0156326 | 0.01735828 | 0.01735828 |
|  | 198.466333 | -0.0061013 | -0.0061013 | 0.01056779 | 0.01056779 |
|  | 19.0333333 | 0.00388331 | 0.00388331 | 0.00672608 | 0.00672608 |
|  | 455.868 | -0.0024674 | -0.0024674 | 0.00427361 | 0.00427361 |
|  | 295.665 | 0.0023396 | 0.0023396 | 0.01474246 | 0.01474246 |
| Day 1 | 267.940333 | -0.1459092 | -0.0648114 | 0.02909371 | 0.03918094 |
|  | 197.882333 | -0.1331089 | -0.3074382 | 0.01417008 | 0.00684715 |
|  | 425.124 | -0.167206 | -0.17867 | 0.00350152 | 0.01024833 |
|  | 218.567333 | -0.2468586 | -0.1183345 | 0.03166042 | 0.01812759 |
|  | 158.978667 | -0.084651 | -0.1668022 | 0.02114879 | 0.01834155 |
|  | 292.672 | -0.0682101 | -0.0880765 | 0.01041673 | 0.02072814 |
|  | 111.107 | -0.0684003 | -0.2393817 | 0.01173793 | 0.01521672 |
|  | 41.367 | -0.2342072 | -0.256783 | 0.02975877 | 0.01089485 |
|  | 134.602333 | -0.1505166 | -0.2655213 | 0.01607685 | 0.01718629 |
|  | 210.735 | -0.0516536 | -0.2303518 | 0.01723254 | 0.01605707 |
| Day 2 | 233.825 | -0.0610635 | -0.0610635 | 0.03067846 | 0.03067846 |
|  | 140.584333 | -0.1354361 | -0.2873418 | 0.00685044 | 0.01464093 |
|  | 302.595333 | -0.2047461 | -0.2047461 | 0.01497081 | 0.01497081 |
|  | 113.751667 | -0.198659 | -0.198659 | 0.01453806 | 0.01453806 |
|  | 89.9246667 | -0.1709642 | -0.1709642 | 0.03567907 | 0.03567907 |
|  | 253.883667 | -0.1032205 | -0.0301937 | 0.00805639 | 0.01423848 |
|  | 118.448667 | -0.2170043 | -0.3249506 | 0.01525382 | 0.00647636 |
|  | 52.81 | -0.2141286 | -0.1879506 | 0.01686422 | 0.02845634 |
|  | 132.842 | -0.3995903 | -0.1918081 | 0.01627582 | 0.02307093 |
|  | 186.460333 | -0.2798243 | -0.1945363 | 0.01028737 | 0.01708143 |

**Supplementary Table 4.** Radial and circumferential stresses for blebbistatin data set.

|  | Distance to edge (um) | Radial stress (Pa) | Circumferential stress (Pa) | (-) Radial stress standard deviation (Pa) | (+) Radial stress standard deviation (Pa) | (-) Circumferential stress standard deviation (Pa) | (+) Circumferential stress standard deviation (Pa) |
| --- | --- | --- | --- | --- | --- | --- | --- |
| Day 0 | 589.337333 | -1.617427 | -1.53564 | 5.830154 | 5.317222 | 6.645397 | 5.760238 |
|  | 138.919 | -1.14694 | -1.117039 | 4.763156 | 4.326811 | 5.422539 | 4.694686 |
|  | 256.589333 | -9.164581 | -9.272991 | 2.197596 | 2.097677 | 2.399856 | 2.306334 |
|  | 470.431667 | -5.06666 | -5.135499 | 3.821231 | 3.854714 | 4.422385 | 4.421893 |
|  | 418.662 | -0.221587 | -0.193348 | 6.961076 | 6.206786 | 7.976663 | 6.779683 |
|  | 611.276 | -6.552472 | -6.61721 | 5.885485 | 5.487462 | 6.57209 | 6.176144 |
|  | 198.466333 | -2.594393 | -2.634016 | 3.349753 | 3.308872 | 3.843778 | 3.649243 |
|  | 19.0333333 | 1.383025 | 1.410119 | 1.824517 | 1.765593 | 2.202045 | 1.998116 |
|  | 455.868 | -1.033383 | -1.054274 | 1.313714 | 1.264916 | 1.541055 | 1.414794 |
|  | 295.665 | 0.774753 | 0.810081 | 4.262314 | 3.755217 | 4.913647 | 4.172076 |
| Day 1 | 267.940333 | -57.369712 | -46.043881 | 22.678407 | 20.125705 | 24.784701 | 22.112644 |
|  | 197.882333 | -225.90097 | -265.39404 | 27.329818 | 25.980629 | 29.09932 | 28.444571 |
|  | 425.124 | -125.67903 | -127.85501 | 14.864042 | 14.817605 | 16.789433 | 16.748918 |
|  | 218.567333 | -127.10968 | -105.62886 | 29.076092 | 27.043713 | 25.043378 | 23.088426 |
|  | 158.978667 | -79.788391 | -93.368335 | 18.454378 | 17.15671 | 20.705925 | 19.034733 |
|  | 292.672 | -40.196489 | -42.995932 | 10.514124 | 10.03789 | 13.476621 | 12.283223 |
|  | 111.107 | -118.26448 | -150.64298 | 20.241123 | 19.099798 | 24.998544 | 23.942445 |
|  | 41.367 | -242.01016 | -247.00051 | 43.390912 | 40.622641 | 39.940205 | 37.205777 |
|  | 134.602333 | -189.86537 | -213.88999 | 33.3809 | 31.325827 | 37.623777 | 35.295874 |
|  | 210.735 | -102.94193 | -135.80386 | 20.372866 | 18.422001 | 24.571439 | 22.64755 |
| Day 2 | 233.825 | -29.461782 | -29.561393 | 15.18428 | 13.624754 | 16.69375 | 14.927409 |
|  | 140.584333 | -203.51195 | -236.34333 | 30.316255 | 28.486122 | 36.13435 | 34.168667 |
|  | 302.595333 | -167.4361 | -167.49788 | 26.342316 | 25.133887 | 27.743775 | 26.001236 |
|  | 113.751667 | -157.93236 | -158.04869 | 24.702411 | 22.913386 | 25.954504 | 24.12351 |
|  | 89.9246667 | -122.48514 | -122.62976 | 40.798365 | 34.944301 | 43.514874 | 37.508318 |
|  | 253.883667 | -32.312682 | -22.770725 | 6.839203 | 6.282639 | 7.3087 | 7.102654 |
|  | 118.448667 | -325.68243 | -352.16013 | 37.930289 | 36.787076 | 38.195174 | 36.990667 |
|  | 52.81 | -158.38726 | -153.76607 | 35.760994 | 32.171951 | 39.741778 | 35.530578 |
|  | 132.842 | -313.57802 | -268.79854 | 55.496495 | 52.042927 | 54.0324 | 50.381177 |
|  | 186.460333 | -209.06344 | -192.22974 | 31.197826 | 30.365373 | 32.403349 | 31.062419 |

**Online Methods.**

**Polyacrylamide biomaterial formulations.** Polyacrylamide (PAA) hydrogel formulations were prepared with the following acrylamide (Bio-rad, 1610140) to bisacrylamide (Bio-rad, 1610142) ratios (see table below), as determined by shear rheology of bulk samples. To polymerize 1 mL of pre-polymer mixture, 100 μL of 1% w/v ammonium persulfate (APS; Bio-rad, 1610700) in phosphate buffered saline (PBS) and 1.5 μL of tetramethylethylenediamine (TEMED; Sigma-Aldrich, T7024) were added to initiate and catalyze the polymerization reaction. 1 μL of 10% w/v fluorescein o-methacrylate (Sigma-Aldrich, 568864) in dimethyl sulfoxide (DMSO) was added to the pre-polymer mixture for the synthesis of fluorescent hydrogels.

| Shear modulus (Pa) | 60 | 400 | 4000 | 7500 |
| --- | --- | --- | --- | --- |
| Acrylamide (wt %) | 3.0 | 3.0 | 7.5 | 7.5 |
| Bisacrylamide (wt %) | 0.06 | 0.10 | 0.06 | 0.24 |

**Microfluidic droplet generator.** A microfluidic droplet generator system consisting of a pulled circular micropipette inserted into a square glass tube^1^ (Supplementary Figure 6) was used to rapidly create hydrogel microspheres of relatively uniform size. Droplets of PAA pre-polymer with the ammonium persulfate initiator and fluorescein-methacrylate monomers were generated in kerosene with 6% w/v polyglycerol polyricinoleate (PGPR 4150). The TEMED catalyst was added downstream of the droplet generation site, and hydrogel microspheres were allowed to polymerize in a kerosene bath. The surfactant-rich kerosene was first replaced with surfactant-free kerosene through multiple centrifugation steps, before being displaced with PBS and allowed to swell. The process enables the rapid production of a large number of uniform hydrogel microspheres, but the large swelling ratio (Supplementary Figure 7) for soft gels magnifies even very slight variations in droplet size.

**Stirred emulsion hydrogel droplet formation.** Polyacrylamide hydrogel microspheres were fabricated using a water-in-oil stirred emulsion technique. All solutions were prepared in clean glass test tubes (Fisher, S63288) capped with rubber septum stoppers (Fisher, FB68681). PAA pre-polymer components were syringe filtered through 0.22 μm pore size nylon filters (Fisher Scientific, 09719C) and purged with N_2_ for 15 minutes. The oil phase, kerosene (Sigma-Aldrich, 329460) with 6% w/v PGPR 4150 surfactant (Palsgaard, 90415001) was purged with N_2_ for 30 minutes. PAA pre-polymer components were syringe injected into the oil phase, and the emulsion was vortex mixed at maximum speed for 10 seconds, generating microspheres ranging from 10 to 100 μm in diameter. The emulsion was magnetically stirred at low speed for 30 minutes while the PAA microspheres polymerized.

**Microsphere recovery and functionalization.** Kerosene with 6% w/v PGPR 4150 surfactant was first replaced with surfactant-free kerosene, and then recovered in PBS through multiple centrifugation steps. Recovered microspheres in PBS are no longer visible in the aqueous phase by eye as there is little polymer content. Microspheres were sterilized under UV light for 45 minutes, and swelled overnight at 4°C. Microspheres were resuspended in 0.05 mg/mL Sulfo-SANPAH (G-Biosciences, BC38) in PBS and irradiated under UV light for 4 minutes to activate the cross-linker. Microspheres were rinsed with PBS, resuspended in 0.05 mg/mL collagen I (VWR, CACB354231) in PBS, and stored overnight at 4°C. Collagen I coated microspheres were resuspended in PBS and stored at 4°C until used for multicellular spheroid (MCS) culture studies. Collagen I functionalization was verified with anti-collagen I mouse primary antibody (Abcam; ab6308) and goat anti-mouse secondary antibody tagged with Alexafluor 594 (Abcam; ab150116). To account for batch to batch variability, one batch of microspheres were used for all MCS experiments.

**Shear rheometry.** Mechanical characterization of bulk polyacrylamide hydrogels was performed using a parallel plate, strain controlled shear rheometer (Anton-Paar, MCR 302). 12 mm glass coverslips were placed in a shaking bath of 0.4% v/v 3-(trimethoxysilyl)propyl methacrylate (MPS; Sigma-Aldrich, M6514) in acetone for 5 minutes, followed by an acetone wash for 5 minutes. PAA pre-polymer mixture was sandwiched between 2 MPS treated coverslips and polymerized to produce hydrogel disks of 1 mm thickness. PAA hydrogel disks were placed in a shaking bath of PBS to swell overnight and to remove non-polymerized monomer. PBS volume was replaced 3 times over the course of 3 hours. Excess PBS was dried off the top and bottom of the hydrogel disks and adhesively fixed between the rheometer plates. The storage modulus was measured at 10% strain from 0.001 to 10 Hz, which was verified to be within the linear elastic regime by an amplitude sweep from 1 to 80% strain at 1 Hz.

**Osmotic pressure measurement of MSG rigidity.** A long-chain dextran solution (500 kDa; www.dextran.ca) was used to extract MSG modulus from osmotic pressure-induced deformations. The system was first calibrated against a bulk disk-shaped polyacrylamide hydrogel (diameter = 13 mm) previously characterized by shear rheometry (as reported in Supplementary Figure 8). A 100 mg/mL 500 kDa dextran solution was used to exert an osmotic pressure on the bulk gel disk and the resulting radial deformation was measured. To determine the osmotic pressure created by the dextran solution, a two-dimensional axisymmetric finite element model was constructed in COMSOL v.5.3.1.201 (Comsol Inc.; Burlington, MA, USA), simulating isotropic pressure-induced deformation of the cylindrical disk-shaped sample. Selection of hyperelastic material properties and meshing considerations are presented in the finite element methods section. A parametric sweep of applied pressure and the resulting strain (Supplementary Figure 10d) was used to determine that 100 mg/mL dextran solution exerts 67 Pa of isotropic pressure on the hydrogel surface. Using this pressure, a parametric sweep of MSG material properties was conducted to determine the relationship between material shear modulus and resultant deformation (Supplementary Figure 10e). MSGs exposed to 100 mg/mL dextran solution will deform to a certain extent, and this curve relates the experimental observation of deformation with the apparent shear modulus of the MSG material. Experimentally, osmotic pressure induced deformation of MSGs occurred rapidly, and microspheres equilibrated within 30 minutes prior to making deformation measurements. Microspheres left in dextran solution for an additional 3 hours confirmed that dextran did not permeate through the polyacrylamide microspheres as no additional deformations were observed (Supplementary Figure 10c). Using this technique, the apparent modulus of the following MSGs were determined: uncoated; coated in collagen I; coated in collagen I, cultured and released from spheroid cultures. Measurement errors of 1 pixel in the images result in errors in estimating the apparent shear modulus of MSGs by ± 7 Pa. The standard deviation in our most varied case (MSGs released from spheroids) was ± 5.3 Pa, suggesting that MSG material properties can be considered constant across each microsphere.

**Characterization of Poisson’s ratio.** Polyacrylamide hydrogel strings were polymerized in 1.3-1.6 mm internal diameter glass tubes (Kimble-Chase, 34500 99) that had been pre-treated with Aquapel (www.aquapel.com) to prevent hydrogel attachment to the inside glass surface. Once polymerized, the hydrogel strings were released and hydrated in PBS for 24 hours prior to mechanically stretching the strings. Hydrogel strings were stretched and observed under a fluorescent stereo microscope (Olympus SZX16) with a LED blue/green light (Crime-Lite 2) and yellow filter. Images were captured with a trinocular-mounted Canon Rebel EOS camera. Changes in string length and corresponding changes in diameter were measured using image analysis, and used to calculate the Poisson’s ratio based on the equation: ν = -ε_diameter_/ε_length_

**Finite element analysis of microsphere deformation.** Two-dimensional axisymmetric finite element models of the MSGs were developed in COMSOL v.5.3.1.201 (Comsol Inc.; Burlington, MA, USA) for the purpose of quantifying axial and radial stresses associated with experimentally-observed MSG deformation. Non-linear stress-strain behavior was captured by simulating the bead as a Neo-Hookean material with Lamé parameters calculated from experimentally-determined shear modulus and Poisson’s ratio values (standard simulations performed with ν = 0.3, G = 60 Pa, or Lamé 1 = 60 Pa, Lamé 2 = 90 Pa). MSG compression and expansion along the axial and radial directions were simulated with strain conditions applied to the MSG solid domain. The radial and axial stresses resulting from the simulated bead deformation were uniform through the MSG for both axial and radial stresses, and read directly from the simulations. A free quad mesh was used and optimized to ensure that the coefficient of variation was less than 1% (mesh element size was 2% spherical diameter; mesh element quality maintained above 0.8 for all strain combinations). A dual parametric sweep of axial and radial strains was performed to capture all possible bead deformation combinations. The stress-strain results were then assembled in MATLAB R2017b (The MathWorks, Inc.) into two surface/contours plots using a piecewise linear interpolation fit. Multicellular spheroid MSG deformation data was then processed using the interpolation fit to obtain the associated axial and radial stresses. Additional parametric sweeps were performed for the Poisson’s ratio to demonstrate the effect of compressibility on the MSG’s ability to read-out stresses (ν = 0, 0.3, 0.499, results in Supplementary Figure 12), and for variations in the apparent shear modulus of MSGs to assess for MSG sensitivity. A deviation of ± 3.5 Pa in the shear modulus results in a ± 5.77% error on the stress measurement (Supplementary Figure 16).

**Monte Carlo analysis of error in MSG deformation.** To obtain an accurate estimate of the error associated with MSG stress measurements, a Monte Carlo error estimation method was employed in MATLAB R2017b (The MathWorks, Inc.) using a script modified to be compatible with piecewise linear interpolation fit functions. MSG dimensions were measured repeatedly in ImageJ and the standard deviation of these measurements was used as the basis for stated errors in measurement precision. For each MSG deformation datapoint, the mean ± standard deviation in axial and radial measurements was used to generate 10,000 random data points, assuming a normal Gaussian probability distribution function for both axes. These 10,000 randomly generated strain values were then each converted to stresses using the piecewise linear interpolation fit (based on data in Fig. 2e, f), to obtain a probabilistic distribution of axial and radial stress. The resulting mean stress value and their 95% confidence intervals were used estimate the precision error associated with measurement of deformation in each MSG datapoint (Fig. 4a-d; Supplementary Figure 16).

**Cell Culture.** HS-5 fibroblasts (ATCC, CRL-11882) were cultured in Dulbecco’s Modified Eagle’s medium (DMEM; Fisher Scientific, 11995073) supplemented with 10% fetal bovine serum (FBS; Fisher Scientific, SH3039603) and 1% antibiotic-antimycotic (Fisher Scientific, 15240062) at 37°C and 5% CO_2_. The medium was changed every 2-3 days. Cells were passaged at 90% confluency.

**Multicellular spheroid cultures.** Multicellular spheroid (MCS) cultures were fabricated using an aqueous two-phase droplet printing technique by an automated liquid handler^2^. The 2 aqueous phases, poly(ethylene glycol) (PEG) and dextran were prepared as follows. 35 kDa PEG (Sigma-Aldrich, 94646) at a concentration of 6% w/v in supplemented DMEM was sterile filtered through a 0.22 μm pore size sterile filter cup and diluted with sterile reverse osmosis (RO) water at a 9:1 (PEG:water) ratio. 500 kDa dextran (www.dextran.ca) at a concentration of 20% w/v in sterile RO water was sterilized under UV light for 45 minutes. Solution were stored at 4°C when not in use. To generate MCS, HS-5 fibroblasts, passaged at 90% confluency, were centrifuged at 200 g for 5 minutes and resuspended at a final concentration of 6x10^7^ cells/mL in supplemented DMEM containing 15% v/v dextran solution. 50 μL of PEG solution was robotically dispensed into each well of a round bottom 96-well plate by an automated liquid handler (Gilson, Pipetmax). 96-well plates were pre-treated with 0.2% w/v Pluronic F-108 (Sigma-Aldrich, 542342) for 1 hour to prevent cell attachment to the bottom of the plate, rinsed with RO water, and dried. 1 μL of cell suspension, containing PAA microspheres at a concentration of ~1 microsphere/μL, was robotically dispensed into each well. The plate was incubated at 37°C in a shaking incubator at 50 rpm for 30 minutes to form spherical droplets. 50 μL of supplemented DMEM was added to each well, and incubated at 37°C and 5% CO_2_ over 2 days to form tightly compacted MCS ~800 μm in diameter. As this protocol involves the handling of micro-volumes of liquid, the use of an automated liquid handler is essential to generate MCS that are reproducible in size. To inhibit contractility, MCS cultures were treated by adding blebbistatin (stock solutions prepared in DMSO) to the culture media immediately after seeding the cells. MCS treated with 50 μM blebbistatin (nominal concentration; Sigma-Aldrich, 203390) did not compact to the same extent as control cultures (Fig. 3b), or those treated with only the DMSO vehicle (Supplementary Figure 20). To confirm that blebbistatin did not permanently damage the contractile apparatus of the cells, it was washed out of culture, after which the MCS contracted to their baseline sizes (Supplementary Figure 20).

**Stress sensor image analysis.** MCS stress mapping imaging was performed on an inverted fluorescent Olympus microscope (Olympus, IX73) outfitted with an sCMOS Flash 4.0 Camera and Metamorph software (version 7.8.13.0). Microsphere position within MCS and microsphere shape were determined using a 10x/0.30 NA on epifluorescence (Olympus, X-CITE 120 LED) on days 0 (n = 9), 1 (n = 17), and 2 (n = 35) of culture. MCS were then lysed in 1% sodium dodecyl sulfate at 37°C for 1 hour and the sensors imaged to obtain microsphere size under zero stress conditions. Microsphere deformation measurements were performed using ImageJ (NIH). Briefly, the multi-point tool was used to select a minimum of 3 points along the microsphere circumference and fitted with either a circle or ellipse selection. This process was repeated 3 times for each microsphere to obtain an average ± standard deviation of microsphere deformation within each MCS and following release from spheroid cultures. All images were processed at the same brightness and contrast levels. Microsphere position within each MCS was measured as the distance from the center of the microsphere to the nearest edge of the MCS in the x-y plane. This value was compared to the vertical distance from the microsphere to the bottom of the MCS using differences between the focal plane of the microsphere and the MCS. The shorter of the two distances was reported as the distance to the edge.

**Tissue sections.** MCS cultured over 2 days were rinsed with PBS and fixed with 4% w/v paraformaldehyde (Sigma-Aldrich, P6148) in PBS for 30 minutes. MCS were rinsed with PBS to remove remaining paraformaldehyde and stored in PBS at 4°C. Fixed MCS embedded in paraffin wax, sliced into 4 μm thick sections, and mounted onto clean glass microscope slides.

**Immunohistochemical analysis.**  Haeomotoxylin and eosin (H&E) staining was conducted following standard protocols. Cell elongation was measured by quantifying cell length manually from these stained tissue sections using ImageJ. A random selection of cells from the edge and core regions were measured over 3 spheroid sections (n = 30). Antigen retrieval of deparaffinized spheroid sections was performed in boiling 10 mM citrate buffer at pH 6.0 for 10 minutes. Spheroid sections were blocked for 5 minutes with Universal Blocking Reagent (BioGenex) at room temperature. Primary anti-Yap/Taz antibody (Cell Signaling, rabbit monoclonal, 1:100) was diluted in blocking solution, anti-Ki67 (Ventana, rabbit monoclonal) and anti-CAIX (Ventana, rabbit polyclonal) were used as per manufacturer’s instructions. Slides were incubated with primary antibodies overnight at 4°C. Immunohistochemical labeling was detected using SignalStain® Boost IHC Detection Reagent (Cell Signaling) and developed with SignalStain® DAB Substrate Kit (Cell Signaling). Spheroid sections were counterstained with hematoxylin. Stained sections were imaged using Aperio ScanScope XT slide scanner, using 20X objective and analyzed using Aperio Spectrum. Resulting stained sections were reviewed by an experienced comparative pathologist (M.C.G.). IHC Nuclear Staining algorithm was used to quantify the fraction of cells exhibiting varying Yap/Taz nuclear staining intensities and the fraction of cells positive for Ki67 nuclear staining across 3 spheroid layers of the following widths: outer 25 µm, middle 50 µm and the remaining inner core. Positive Pixel Count algorithm was used to quantify the average intensity of all pixels of CAIX staining in 25 µm outer layer and the remaining core. Spatial positions were scaled based on the observed shrinkage of the tissues during the fixation processes (factor of ~2), and reported as dimensions in the original tissue.

**Immunofluorescence analysis.**  Immunofluorescence staining of phospo-myosin was performed following a previously described protocol^3^. Paraffin sections were deparaffinized and boiled in Tris-EDTA buffer (20 mM Tris, 1 mM EDTA, 0.05% Tween-20 at pH 9.0) for 7 minutes in a pressure cooker. Sections were blocked in 10% normal goat serum for 1 hour followed by overnight incubation of primary antibody at 4°C. Sections were incubated for 1 hour with the appropriate secondary antibody conjugated to Alexa546 (1:750 dilution). The primary antibody used was an anti-phospho-myosin light chain 2 (pMLC) (1:200, CST #3671). Actin filaments were stained with 0.1 µM phalloidin for 15 minutes. Confocal imaging was performed on an LSM700 from Zeiss with 20x/0.8 NA or 40x/1.4 NA lenses.

**Second harmonic generation imaging.** SHG images were acquired on a LSM710 Multiphoton (Carl Zeiss Canada) equipped with a Plan-Apochromat 20x/0.8 NA, 800 nm direct-coupled multiphoton laser with or without dispersion compensation – Chameleon (Coherent), and SHG signals from Ch1: 493-630 were recorded with PMT detectors with gain of 750 V.

**Volumetric growth calculations.** Volumetric growth calculations were performed based on Ki67 and H&E sections. All images were analyzed in 3 distinct regions: edge, intermediate zone, and core. H&E images were analyzed for white content to determine the area not occupied by cells. These values were subtracted from the area of each region and divided by the number of nuclei to obtain the area occupied by a single cell, which was then converted to the volume occupied by a single cell assuming spherical geometry. Ki67 results were used to determine the number of dividing nuclei. This value was multiplied by the number of initial nuclei to determine the number of new nuclei following a cycle of cell division. Volumetric expansion was defined as the ratio of the new volume (new total nuclei x volume of a single cell) to the old volume.

**Finite element modeling of tissue sphere formation.** Finite element models of the tissue MCS were constructed in FEBio as a solid sphere containing three layers with distinct mechanical properties^4^. The layer thickness from the surface to the core of the sphere was 50 µm (edge), 100 µm (intermediate zone), and 350 µm (core), respectively. The active contraction of the cell population was represented by a solid mixture material consisting of a neo-Hookean solid component and an isotropic contractile stress component^5^. The compressive neo-Hookean solid allows compaction of the tissue microsphere model under active contraction. Three contraction and stiffness conditions were modeled for the tissue microsphere and the circumferential and radial stresses for these conditions were compared. In the first case, all three layers of the sphere model were assumed to have the same stiffness and undergo the same amount of active contraction (isovolumetric). In the second case, all three layers of the sphere model were assumed to have the same stiffness with only the outermost layer undergoing active contraction (outer contraction; uniform stiffness). In the third case, the stiffness of the outermost layer and the intermediate layer of the sphere model were assumed to be 2 times higher than that of the core layer, with only the outermost layer undergoing active contraction. Tissue MCS geometry was discretized by about 8800 3D quadratic tetrahedral elements capable of large deformation.

**Statistical analysis.** Comparative data analysis of populations was performed without pre-specifying a required effect size. Datasets that were normally distributed, with similar variances between compared groups were analyzed using one-way ANOVA; or ANOVA based on ranks to test for significance in the three layers of the MCS (edge, intermediate zone, and core), and for characterization of MSG apparent shear modulus. Post-hoc pairwise comparisons were conducted using the Tukey’s method. All statistical analyses were performed using SigmaStat 3.5 (Systat Software Inc., San Jose, CA, USA).

**Supplemental References**

1. Chu, L.-Y., Kim, J.-W., Shah, R. K. & Weitz, D. A. Monodisperse Thermoresponsive Microgels with Tunable Volume-Phase Transition Kinetics. *Adv. Funct. Mater.* **17,** 3499–3504 (2007).

2. Ham, S. L., Atefi, E., Fyffe, D. & Tavana, H. Robotic Production of Cancer Cell Spheroids with an Aqueous Two-phase System for Drug Testing. *JoVE J. Vis. Exp.* e52754–e52754 (2015). doi:10.3791/52754

3. Halaoui, R. *et al.* Progressive polarity loss and luminal collapse disrupt tissue organization in carcinoma. *Genes Dev.* **31,** 1573–1587 (2017).

4. Maas, S. A., Ellis, B. J., Ateshian, G. A. & Weiss, J. A. FEBio: Finite Elements for Biomechanics. *J. Biomech. Eng.* **134,** 011005-011005-10 (2012).

5. Ateshian, G. A., Costa, K. D., Azeloglu, E. U., Morrison, I., Barclay & Hung, C. T. Continuum Modeling of Biological Tissue Growth by Cell Division, and Alteration of Intracellular Osmolytes and Extracellular Fixed Charge Density. *J. Biomech. Eng.* **131,** 101001-101001–12 (2009).
